# YB1 modulates the DNA damage response in medulloblastoma

**DOI:** 10.1038/s41598-023-35220-6

**Published:** 2023-05-19

**Authors:** Leon F. McSwain, Claire E. Pillsbury, Ramona Haji-Seyed-Javadi, Sandip Kumar Rath, Victor Chen, Tiffany Huang, Shubin W. Shahab, Haritha Kunhiraman, James Ross, Gabrielle A. Price, Abhinav Dey, Dolores Hambardzumyan, Tobey MacDonald, David S. Yu, Christopher C. Porter, Anna M. Kenney

**Affiliations:** 1 Department of Pediatrics, Emory University, 1760 Haygood Dr., Atlanta, GA 30322 USA; 2 Winship Cancer Institute, Emory University, Atlanta, GA 30322 USA; 3 Department of Biology, Emory University, Atlanta, GA 30322 USA; 4 Department of Microbiology and Immunology, Emory Vaccine Center, Emory University, Atlanta, GA USA; 5 Department of Neurosurgery, Icahn School of Medicine at Mount Sinai, New York, NY 10029 USA

**Keywords:** Paediatric cancer, Cancer, Cell biology, Oncology

## Abstract

Y-box binding protein 1 (*YBX1* or YB1) is a therapeutically relevant oncoprotein capable of RNA and DNA binding and mediating protein–protein interactions that drive proliferation, stemness, and resistance to platinum-based therapies. Given our previously published findings, the potential for YB1-driven cisplatin resistance in medulloblastoma (MB), and the limited studies exploring YB1-DNA repair protein interactions, we chose to investigate the role of YB1 in mediating radiation resistance in MB. MB, the most common pediatric malignant brain tumor, is treated with surgical resection, cranio-spinal radiation, and platinum-based chemotherapy, and could potentially benefit from YB1 inhibition. The role of YB1 in the response of MB to ionizing radiation (IR) has not yet been studied but remains relevant for determining potential anti-tumor synergy of YB1 inhibition with standard radiation therapy. We have previously shown that YB1 drives proliferation of cerebellar granular neural precursor cells (CGNPs) and murine Sonic Hedgehog (SHH) group MB cells. While others have demonstrated a link between YB1 and homologous recombination protein binding, functional and therapeutic implications remain unclear, particularly following IR-induced damage. Here we show that depleting YB1 in both SHH and Group 3 MB results not only in reduced proliferation but also synergizes with radiation due to differential response dynamics. YB1 silencing through shRNA followed by IR drives a predominantly NHEJ-dependent repair mechanism, leading to faster γH2AX resolution, premature cell cycle re-entry, checkpoint bypass, reduced proliferation, and increased senescence. These findings show that depleting YB1 in combination with radiation sensitizes SHH and Group 3 MB cells to radiation.

## Introduction

For decades, the standard of care for medulloblastoma (MB) treatment has consisted primarily of surgical resection and a combination of radiation and cisplatin-based chemotherapy^1^. While patient survival has greatly benefited from this regimen there are significant sequelae that result, including endocrine abnormalities, hearing loss, and neurocognitive decline. Additionally, some MB molecular subgroups, including *TP53*-mutant Sonic hedgehog-activated (SHH) and Group 3, have a substantially worse survival outcome with a higher incidence of relapse^2,3^. In addition to the investigation of small molecule inhibitors targeting genetic and transcriptomic alterations specific to the four subgroups (Wingless—WNT, Sonic Hedgehog (SHH), Group 3 and Group 4), clinical trials have also focused on targeting proteins that mediate resistance to DNA damaging therapies^4^. We previously showed that Yes-Associated Protein (YAP) drives Y-box binding protein 1 (YB1) activity, resulting in Insulin-like growth factor 2 (IGF-2) promoter binding and an autocrine feedback loop which promotes proliferation of cerebellar granular neural precursors (CGNPs) and NeuroD2-SmoA1-derived primary SHH mouse medulloblastoma cells (referred to as MBCs)^5^. Given the overexpression of YB1 across the four molecular-defined MB subgroups compared to non-tumor controls, we sought to determine whether YB1 plays a role in the MB radiation response. The mechanistic properties regulating YB1 cellular localization and functionality are well established but are lacking and inconsistent between cancer models with respect to YB1’s role in response to ionizing radiation (IR). Compared to anatomically matched control brain tissue, YB1 expression is elevated in several types of adult and pediatric brain tumors, including glioblastoma multiforme, ependymoma, anaplastic astrocytoma, and diffuse intrinsic pontine glioma^6^. YB1 can also drive a variety of stemness, metastasis, proliferation, angiogenesis, and drug resistance phenotypes in other cancers, including neuroblastoma, breast, lung, colorectal, and others^6,7^. YB1 nuclear transport appears to be a prerequisite to drive these phenotypes, a mechanism induced by environmental stressors and preceded by serine 102 phosphorylation and c-terminal cleavage^8,9^.

Several groups have emphasized a role for YB1 in the DNA damage response, focusing on direct interactions with DNA or repair proteins. YB1 was shown to mediate strand separation of cisplatin-bound DNA in addition to driving expression of the MDR1 receptor, resulting in cisplatin efflux^10–13^. Additionally, following etoposide or doxorubicin treatment of NIH3T3 cells, the proteolytic YB1 fragment was found to interact with proteins Mre-11 and Rad50 that are responsible for homologous recombination; and, following radiation, YB1 can be phosphorylated by DNA-PKcs to accelerate repair^14,15^. As the more deleterious effects of IR involve repair of double strand breaks (DSBs) through homologous recombination (HR) or non-homologous end joining (NHEJ), any role for YB1 in this process could lead to synergism. While YB1 was found to potentiate PARP1-mediated ribosylation of DNA following IR-induced DSBs leading to PARP inhibitor resistance, in vivo studies have not corroborated these findings, and targeting YB1 could ameliorate the need for PARP inhibition^16^. Additionally, though YB1 was found to colocalize with p53 and Werner Syndrome Protein following UV treatment, colocalization was not observed between YB1 and γH2AX, a marker of chromatin de-condensation proximal to sites of DNA damage^17^. Thus, a direct and functional role for YB1 in the response to IR remains to be seen.

In the present study, we extend our previous findings on YB1 as a driver of proliferation into Group 3 MB and demonstrate the functional consequences of YB1 depletion following IR in SHH and Group 3 MB. We show that YB1 knockdown (KD) cells utilize differential repair pathways and fail to recognize and activate cell cycle checkpoints, resulting in decreased proliferation and increased senescence in YB1-depleted cells.

## Results

### YB1 is expressed across all MB subgroups and overexpression is associated with shortened survival in an SHH primary mouse model

**Figure 1:**
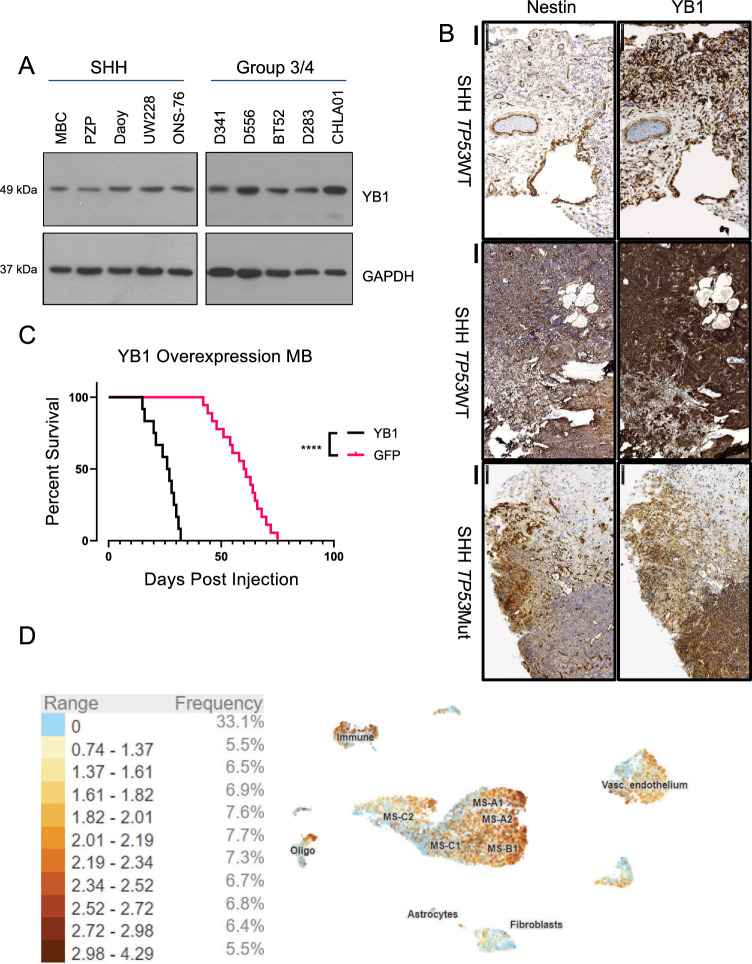
YB1 is expressed across all MB subgroups and overexpression is associated with shortened survival in an SHH primary mouse model (**A**) Immunoblotting cell lysate from SHH cells (MBC (primary NestinD2-SmoA1), Pzp53Med (Ptch-LacZ-p53null), Daoy, UW228, and ONS-76), and group 3/4 cells (D341, D556, BT52, D283, and CHLA01) with GAPDH as control. (**B**) Immunohistochemistry of SHH subgroup samples from both TP53 wild type and mutated patients showing positive staining of both Nestin (Stem marker) and YB1 (Left scale bar = 100 μm, quantification Supp Fig. 1a and b). (**C**) Survival analysis of BL6 mice orthotopically implanted with NestinD2-SmoA1 primary cells following adenoviral overexpression of YB1 (GFP median survival 26.5 days YB1 median survival 60.5 days p < 0.0001). (**D**) UMAP of previously published single cell sequencing analysis of SHH-Math-Cre-SmoM2 showing enrichment of YB1 in numerous cell populations collected from UCSC Cell Browser: active cell cycling (MS-A1 and MS-A2) and progenitor (MS-B1). Expression profile is subdivided into 10 expression ranges apart from no expression and percent of all cells within each range listed on right.

Previously, we have shown that YB1 RNA is elevated across MB subgroups^5,18^. We sought to corroborate YB1 RNA levels with corresponding protein expression data by immunoblotting cell lysates from NeurD2-SmoA1 primary SHH mouse MB cells (MBCs), a *TP53* null PTCH receptor-deficient spontaneous SHH tumor mouse derived MB cell line (Pzp53Med^19^), human SHH MB cell lines (Daoy, UW228, and ONS-76), and human Group 3 and 4 MB cell lines (D341, D556, BT52, D283, and CHLA01). Interestingly, YB1 protein is robustly expressed across all cell lines (Fig. 1a). Immunohistochemistry (IHC) of 3 patient samples from both SHH MB *TP53-*wild type and mutant tumors, whose patients respond poorly to standard of care, shows that YB1 is highly expressed in all samples (Fig. 1b and Supp. Fig. 1a,b)^20^. Given challenges with stable protein knockdown in primary MB mouse models, we chose to transiently overexpress YB1 in NeurD2-SmoA1-derived primary cells followed by orthotopic implantation into the cerebella of p5 mice to determine the role of YB1 on tumor growth in SHH medulloblastoma. Mice implanted with YB1*-*overexpressing MBCs had a median survival of 26.5 days compared to mice injected with GFP control-transduced MBCs, which had a median survival of 60.5 days (*p* < 0.0001) (Fig. 1c). Single cell sequencing of SHH primary MB mouse models published by Riemondy et al. allowed us to further assess a potential role for YB1 in cell subpopulations within the tumor (Fig. 1d)^21^. In the SHH-Math-Cre-SmoM2 primary SHH model, YB1 is elevated across most cell populations, particularly in those representing immune, active cell cycling (MS-A1 and MS-A2), and progenitor (MS-B1) populations. In the MYC-driven p53 dominant negative Group 3 spontaneous mouse model (GP3-Myc-dnP53), YB1 is highly elevated in subpopulations corresponding to active cell cycling (MP-A1, -2) and progenitor (MP-B1, -B2, -B3) profiles compared to differentiated neoplastic subpopulations (MP-C1, -C2) (Supp. Fig. 1c)^22^. In addition to data showing worse survival in mice harboring YB1-overexpressing tumors and robust YB1 protein express across all subgroups, the single cell sequencing expression profiles suggest that YB1 inhibition in stem or progenitor-like populations previously implicated in driving relapse could sensitize tumor cells to radiation, resulting in improved therapeutic response.

### YB1 depletion results in differential cell cycling and reduction of aberrant nuclear morphology following radiation

**Figure 2:**
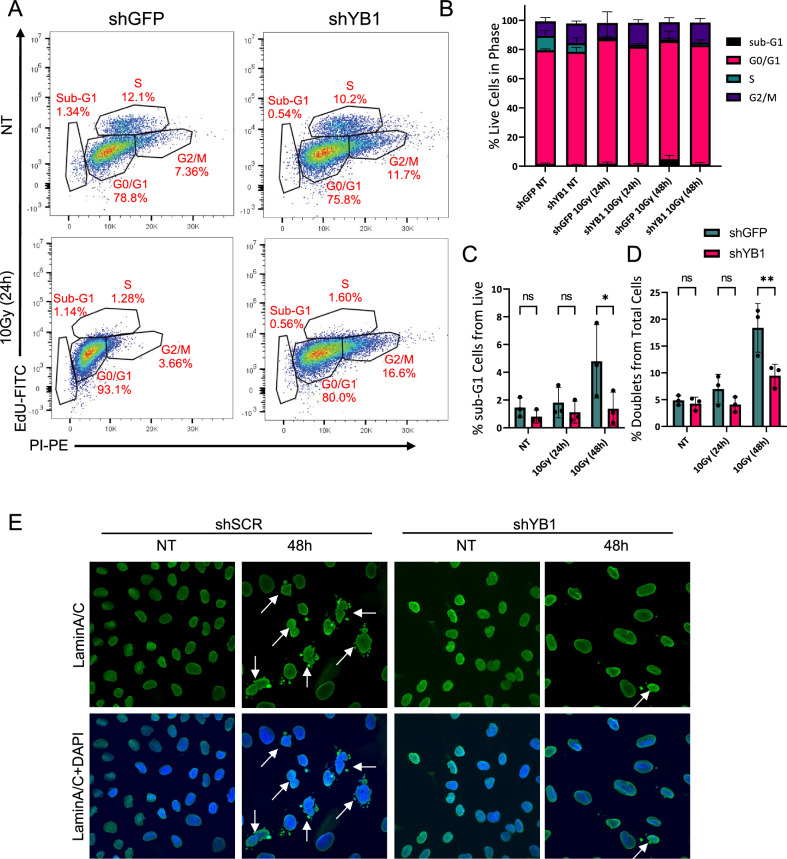
YB1 Knockdown results in differential cell cycling and reduction of aberrant nuclear morphology following radiation (**A**) ONS-76 shGFP and shYB1 cells were untreated (NT) or treated with 10Gy and analyzed for cell cycle phase proportions (sub-G1, G0/G1, S, and G2/M) at 24 (shown) and 48 h using EdU and PI. Quantification of cell cycle phase distribution (SD and Means Supp Fig. 2, n = 3 ) (**B**). At 48 h post-irradiation, shYB1 shows significantly lower proportions of cells in sub-G1 (shGFP vs shYB1 95% CI = 0.36–6.47 p = 0.0275, n = 3) (**C**) and cells appearing in doublets (shGFP vs shYB1 95% CI = 3.303–14.56 p = 0.0026, n = 3) (**D**) (doublets excluded from cell cycle analysis). (**E**) shSCR and shYB1 non-treated and treated with 10Gy were stained with LaminA/C and DAPI 48 h after 10Gy irradiation. shSCR cells demonstrate more aberrations in nuclear morphology (UW228 and additional images Supp Fig. 3).

To characterize the result of YB1 depletion on cell cycle distribution up to 48 h (h), human SHH MB ONS-76 short-hairpin control (shGFP) and YB1 knockdown (KD) (shYB1) cells were exposed to 10 Gray (Gy) IR at 24 and 48 h post-plating and cultured for 72 h prior to analysis via an EdU incorporation assay (Fig. 2a and Supp. Fig. 2). The cell cycle distribution between non-treated YB1 KD and control cells was very similar, though YB1 KD cells had a modest decrease in percentage of cells in S-phase and increase in percentage of cells in G2/M phase compared to control, consistent with a demonstrated role for YB1 in the transition to and completion of mitosis^23^. Following radiation, a greater proportion of control cells entered the terminal sub-G1 phase by 48 h compared to KD cells (*p* = 0.0275, Fig. 2b,c). At 48 h, cells showed significant differences not only in cell cycle distribution but also in nuclear morphology. Control cells had an increase in proportions of cells in doublets at 48 h post-radiation (*p* = 0.0026, Fig. 2d). To confirm that this doublet morphology was not a technical artifact of flow cytometric analysis, we stained cells for LaminA/C in both ONS-76 and UW228, a *TP53*-mutated cell line (Fig. 2e and Supp. Fig. 3). And while UW228 showed similarities in nuclear morphology of control irradiated cells to ONS-76, there were no statistically significant differences in cell cycle distribution between control and YB1 depleted irradiated cells, potentially due to differences in basal p53 levels between these cells. The elevated nuclear fractionation indicated by the LaminA/C staining in control irradiated cells compared to YB1-depleted irradiated cells at 48 h following radiation in both ONS-76 and UW228 suggests these cells are experiencing mitotic catastrophe and incomplete cytokinesis^24^. While there are many potential explanations, differences in cell cycle percentages paired with observed changes in nuclear morphology of irradiated cells could indicate differences in cell cycle regulation or repair pathway choice. Given YB1 is known to suppress the activity of p16 and p53^25–27^, our observations could suggest a failure to signal non-viable levels of incomplete repair as a result of YB1 loss.

### YB1 depletion results in differential γH2AX resolution and CHK2 phosphorylation in SHH and Group3 MB cells

**Figure 3:**
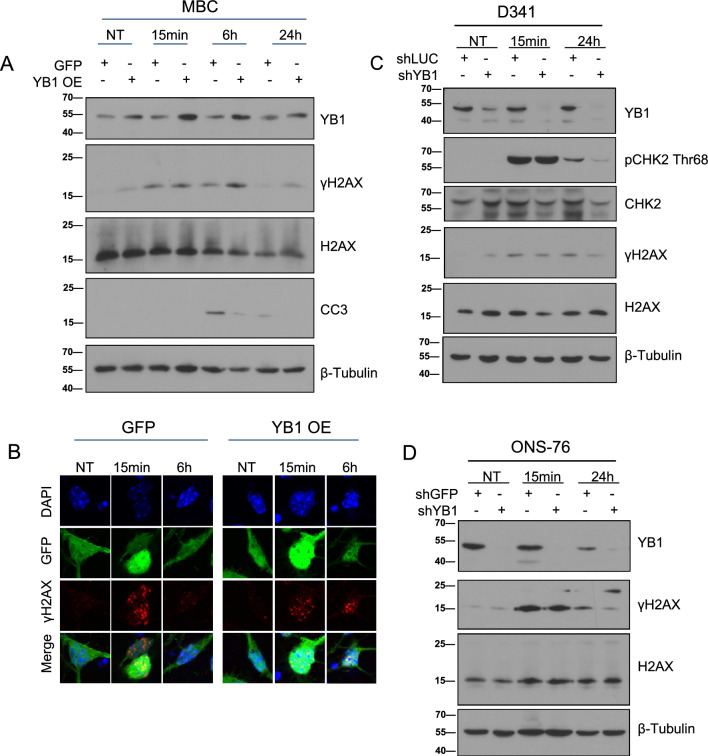
YB1 depletion results in differential γH2AX resolution and Chk2 phosphorylation in SHH and Group3 medulloblastoma cells (**A** and **B**) MBCs plated for 24 h prior to infection with either control or YB1 overexpressing adenovirus. Following 48 h incubation cells were irradiated with 2Gy and either lysed (immunoblotting) or fixed prior to staining (immunofluorescence). (**C**) D341 shLuc (control) and shYB1 cells irradiated with 5Gy (Expanded western Supp Fig. 5). (**D**) ONS-76 shGFP (control) and shYB1 cells irradiated with 10Gy (Additional replicates Supp Fig. 5).

Differences in cell cycle ratios and nuclear morphology between control and YB1 KD cells suggest that YB1 may influence DNA repair pathway choice, with YB1 driving a more time-consuming repair, in addition to a potential role in regulating transition from G2/M or regulation of p16 or p53. To determine whether YB1 plays a more direct role in the DNA damage response to IR, we performed cellular fractionation and radiation time courses in the NeuroD2-SmoA1 primary MBCs. We exposed MBCs to 2 Gy radiation and assessed cytoplasmic, nuclear, and chromatin fractions to determine YB1 intracellular distribution by immunoblotting (Supp. Fig. 4a). We found that YB1 is robustly distributed between all subcellular fractions, and there was no observed difference in nuclear or chromatin fraction-localized YB1 levels between irradiated and non-irradiated cells up to 30 min post-radiation. Due to toxicity of lentivirus in combination with radiation in primary cells, we irradiated YB1-overexpressing MBCs. Following 6 h of recovery from initial exposure, γH2AX persists in YB1-overexpressing cells compared to GFP- control MBCs, and YB1-overexpressing cells show lower levels of cleaved caspase 3 (CC3), a marker of cell death (Fig. 3a and Supp. Fig. 4b). When we performed immunofluorescence (IF) with these cells, we further observed γH2AX foci persistence in YB1-overexpressing cells compared to control cells data (Fig. 3b and Supp. Fig. 4c). We chose to confirm these effects in control or shYB1 ONS-76 and D341 cell lines, representing human models of SHH and Group3 MB respectively. 24 h after IR exposure, both cell lines showed higher γH2AX persistence in control cells compared to YB1 KD cells (Fig. 3c,d and Supp. Fig. 5a–d). Human SHH MB Daoy (*TP53* mutant) cells also showed lower γH2AX at 6 h and 24 h in YB1 KD cells compared to controls (Supp. Fig. 4c). Moreover, in D341 cells, phospho-Chk2 intensity was higher at 24 h compared to YB1 KD cells (Fig. 3c). The elevated γH2AX and phospho-Chk2 in cells expressing YB1 is consistent with the reduced viability and accumulation of control-irradiated cells in sub-G1 of ONS-76 48 h following eradiation (Fig. 2a–c). The unresolved γH2AX present in control but not YB1-deficient cells at 24 h persisted regardless of cell line or control shRNA construct utilized, as seen in later experiments where γH2AX again was present at higher levels at 24 h in ONS-76 scramble controls. Following radiation, this observed correlation between γH2AX resolution and YB1 expression, paired with our observed differences in cell cycle re-entry at 24 h, is suggestive of immediate differences in repair pathway choice that may contribute to genomic instability or genomic rearrangements, which in turn could affect cell viability and therapeutic response.

### YB1 depletion results in accelerated DSB and SSB repair, γH2AX resolution, and a lack of RPA32 phosphorylation at Serines 4/8

**Figure 4:**
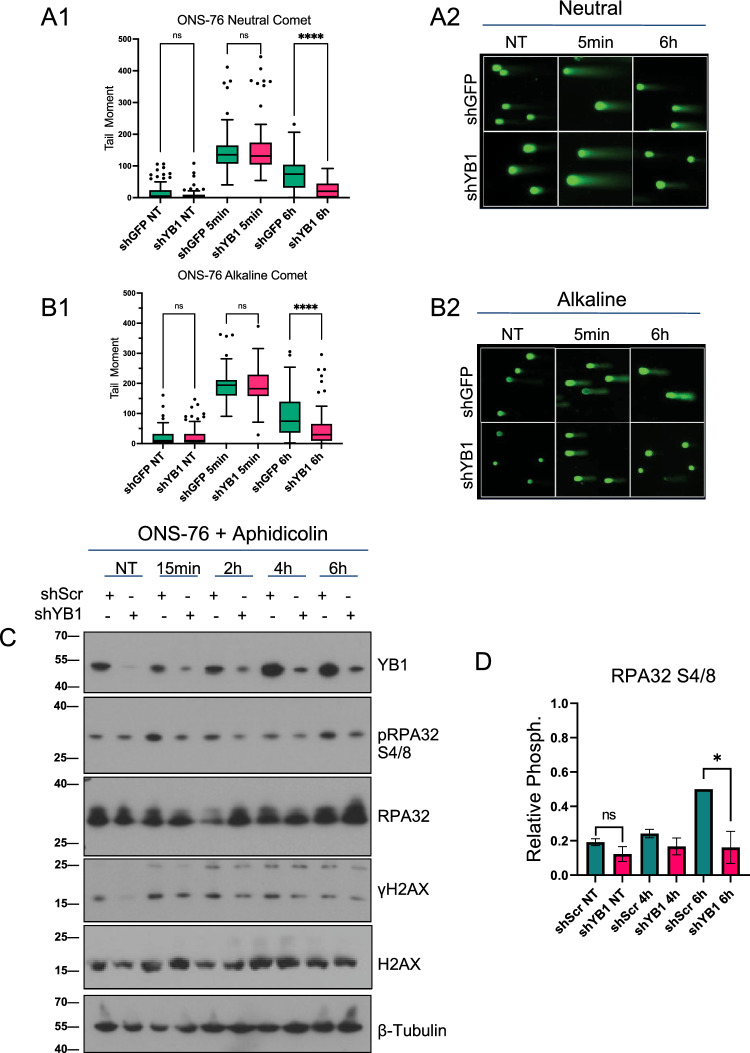
YB1 depletion results in accelerated physical repair, γH2AX resolution, and lack of RPA32 phosphorylation at Serines 4/8 (**A1** and **A2**) Neutral comet assay tail moment of ONS-76 shGFP and shYB1 treated with 10Gy showing non-significant differences in damage accumulation (shGFP vs shYB1, shGFP 95% CI = 171–164.6 shYB1 95% CI = 135–178.3 p > 0.9999, Kruskal-Wallis test, n = 3) and significant differences in damage resolution at 6 h (shGFP vs shYB1, shGFP 95% CI = 62.86–86.59 shYB1 95% CI = 19.06–30.49 p < 0.0001, Kruskal-Wallis test, n = 3). (**B1** and **B2**) Alkaline comet assay tail moment of ONS-76 shGFP and shYB1 treated with 10Gy showing nonsignificant differences in damage accumulation (shGFP vs shYB1, shGFP 95% CI = 180.5–201.5 shYB1 95% CI = 183.3–207.0 p > 0.9999, Kruskal-Wallis test, n = 3) and significant differences in damage resolution at 6 h (shGFP vs shYB1, shGFP 95% CI = 80.1–105.1 shYB1 95% CI = 37.84–57.05 p < 0.0001, Kruskal-Wallis test, n = 3). (**C**) Synchronization of ONS-76 with Aphidicolin for 24 h prior to radiation time course at 10Gy (Additional replicates Supp Fig. 6). (**D**) Densitometry of pRPA32 S4/8 shows consistent elevation in shGFP cells compared to shYB1 6 h post-IR (shGFP vs shYB1 95% CI = − 0.24–0.56 p = 0.0223, Ratio paired t-test, n = 3, internal normalization to shGFP 6 h).

Previous publications have linked YB1 and HR through interactions between a proteolytic fragment of YB1 and proteins Rad50 and Mre-11, components of the MRN complex (Mre-11/Rad50/NBS1), following chemotherapeutic exposure^14^. In our models, YB1 proteolytic processing and its effects on nuclear localization are unclear. However, given that we detect full length YB1 in the nuclear and chromatin fractions and our observations of differential γH2AX beyond initial accumulation at 15 min, we hypothesized that irradiated cells deficient in YB1 perform less HR, a slower process than NHEJ, and thus resolve DSBs, SSBs, and γH2AX foci more rapidly compared to control irradiated cells^28,29^. We analyzed single cell DNA damage resolution using neutral and alkaline comet assays to understand single and double stranded break accumulation and resolution. Under both neutral (Fig. 4a1,a2) and alkaline conditions (Fig. 4b1,b2), shYB1 cells resolve damage faster than irradiated control cells after accumulating similar levels of damage. To further test the hypothesis that YB1-deficient cells preferentially utilize a more rapid repair pathway, we incorporated an alternative scramble shRNA control given potential concerns about off target effects of sh controls and performed cell synchronization using aphidicolin, which arrested cells in S-phase. Following 10 Gy irradiation in aphidicolin synchronized ONS-76 cells (Fig. 4c and Supp. Fig. 6), YB1 KD cells resolved γH2AX faster than control-irradiated cells starting at 4 h and continuing into 6 h, a phenotype conserved in UW228 (Supp. Fig. 7). Interestingly, by 6 h post-radiation, the majority of γH2AX is resolved but re-emerges in control-irradiated cells at 24 h, a phenotype conserved across cell lines regardless of short-hairpin construct used (Figs. 3, 4). Indeed, cells entering sub-G1 or initiating apoptosis can express γH2AX as part of apoptotic bodies^30,31^. This phenotype was made apparent in ONS-76 cells by the re-emergence of γH2AX in control-irradiated cells at 24 h concomitant with an increase in sub-G1 control irradiated cells both 24 and 48 h following radiation (Fig. 2). Finally, to begin our assessment of a potential decrease in HR-mediated repair, we probed RPA32 at Serines 4/8 in aphidicolin-synchronized ONS-76 cells (Fig. 4c,d and Supp. Fig. 6). RPA32 was significantly more phosphorylated at 6 h in irradiated synchronized control cells compared to YB1-silenced cells across replicates. Given RPA32 can be phosphorylated in response to replication stress and is implicated in binding ssDNA prior to Rad51 during HR strand invasion, a lack of RPA32 phosphorylation in shYB1 cells suggests a lower degree of replication stress and HR following radiation treatment^32,33^. The increased pRPA32, delayed γH2AX resolution, and re-emergence of γH2AX at 24 h in control-irradiated cells further supports that YB1 could be driving an alternate repair mechanism.

### YB1 depleted cells accumulate less RAD51 and more TP53BP1 nuclear bodies during and after S-Phase repair

**Figure 5:**
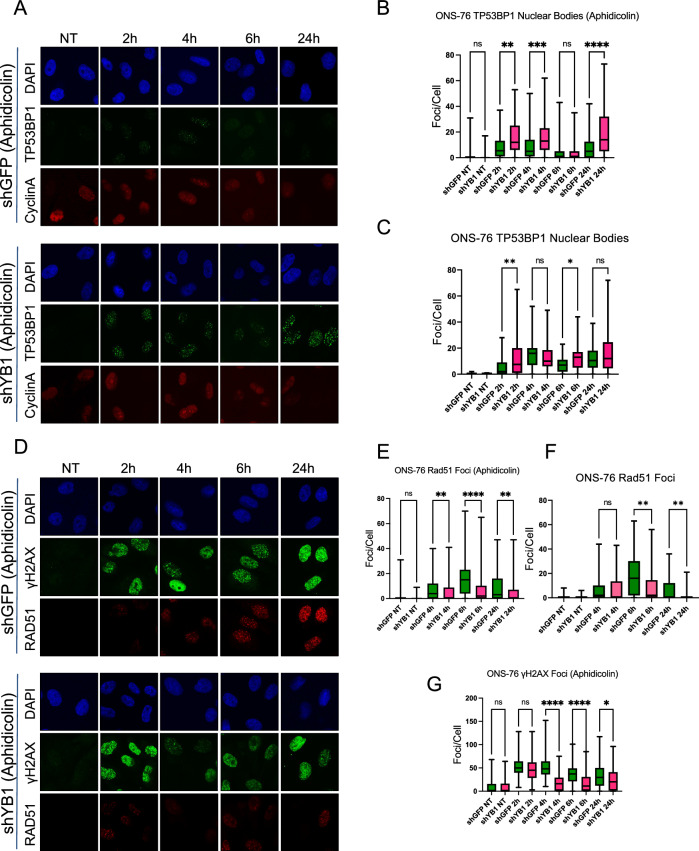
YB1 depleted cells accumulate less RAD51 and more TP53bp1 foci during and after S-Phase repair. (**A** and **B**) Aphidicolin S-phase synchronization of shGFP and shYB1 ONS-76 24 h prior to radiation at 10Gy results in greater TP53BP1 accumulation in shYB1 cells that is sustained until 6 h and reappears at 24 h (2 h Mean rank diff. = −149.0 p = 0.0045, 4 h mean rank diff. = −168.9 p < 0.0004, 6 h Mean rank diff. = 35.54 p > 0.9999, 24 h mean rank diff. = −208.7 p < 0.0001, Kruskal-Wallis test, n = 3). (**C** and Supp Fig. 8a) Non-Synchronized ONS-76 exposed to 10Gy results in greater TP53BP1 accumulation in shYB1 cells that is sustained until 24 h (2 h Mean rank diff. = −121.0 p = 0.0080, 4 h Mean rank diff. = 52.42 p = 0.7142, 6 h Mean rank diff. = –117.1 p = 0.0117, 24 h Mean rank diff. = −34.30 p > 0.9999, Kruskal-Wallis test, n = 2). (**D** and **E**) Aphidicolin S-phase synchronization of shGFP and shYB1 ONS-76 24 h prior to radiation at 10Gy results in reduced RAD51 accumulation in shYB1 cells up to 6 h and at 24 h (4 h Mean rank diff. = 141.0 p = 0.0018, 6 h Mean rank diff. = 234.9 p < 0.0001, 24 h Mean rank diff. = 135.6 p = 0.0087, Kruskal-Wallis test, n = 3). (**F** and Supp Fig. 8b) Non-Synchronized ONS-76 exposed to 10Gy results in reduced RAD51 accumulation in shYB1 cells up to 6 h and at 24 h (4 h Mean rank diff. = 41.01 p = 0.8115, 6 h Mean rank diff. = 144.3 p = 0.0007, 24 h Mean rank diff. = 176.9 p = 0.0031, Kruskal-Wallis test, n = 2). (**D** and **G**) Aphidicolin S-phase synchronization of shGFP and shYB1 ONS-76 24 h prior to radiation at 10Gy results in faster γH2AX resolution up to 6 h that reappears in shGFP control at 24 h (NT Mean rank diff. = 14.48 p > 0.9999, 2 h Mean rank diff. = 82.26 p = 0.2664, 4 h Mean rank diff. = 406.7 p < 0.0001, 6 h Mean rank diff. = 245.9 p < 0.0001, 24 h Mean rank diff. = 125.3 p = 0.0205, Kruskal-Wallis test, n = 3).

Differences in γH2AX, RPA32 phosphorylation, and DSB and SSB repair kinetics are suggestive of a repair pathway switch following ionization radiation damage. We further investigated the activation of HR or NHEJ specific proteins in asynchronous and synchronized ONS-76 cells through IF. Since TP53BP1 is known to be involved early in the DNA repair signaling process to inhibit end resection and promote NHEJ^34^, we sought to investigate its nuclear localization following radiation. Compared to the 10 Gy irradiated control cells, irradiated YB1-depleted cells show greater levels of TP53BP1 nuclear bodies in synchronized cells, with greatest levels observed at 2 h (Fig. 5a,b). RIF1, which binds to TP53BP1 to facilitate NHEJ^35^, is also enriched in YB1-depleted cells at 2 h post-radiation (Supp. Fig. 9). Following synchronization, however, YB1-depleted cells fail to accumulate RAD51 foci at levels comparable to control irradiated cells at 6 h (Fig. 5d,e). Elevated TP53BP1 and reduced Rad51 are conserved in non-synchronized irradiated YB1-depleted cells (Fig. 5c,f and Supp. Fig. 8). Finally, γH2AX foci resolve faster in synchronized-irradiated YB1-depleted ONS-76 cells (Fig. 5g), which is consistent with western blotting (Fig. 4c).

### YB1 depletion results in greater canonical NHEJ and lower HR

**Figure 6:**
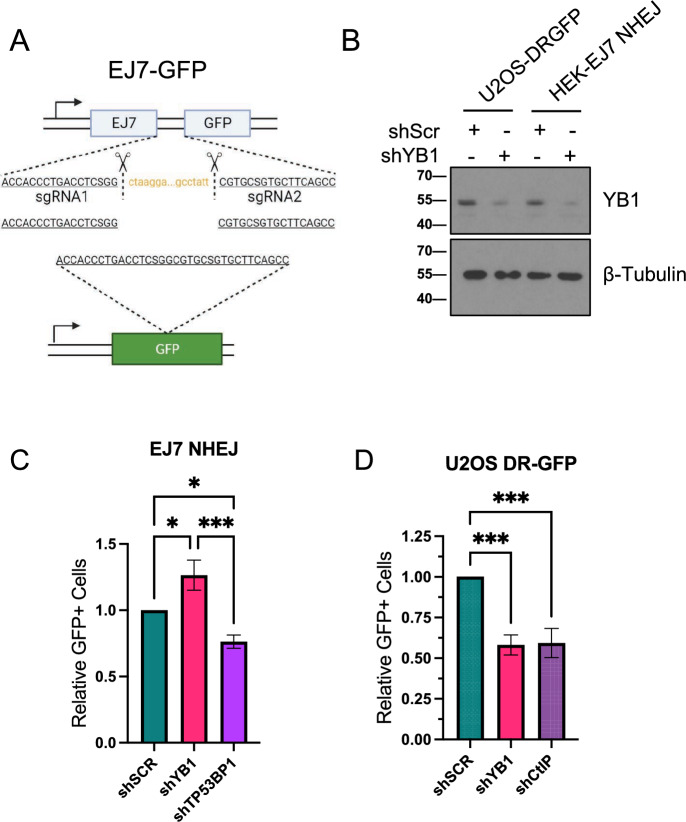
YB1 depletion results in greater canonical NHEJ and lower HR (**A**) Schematic of distal EJ without indels assay whereby two sgRNAs are co-transfected with SCEI to generate blunt ends repairable through cNHEJ to restore GFP expression. (**B**) Western blot of YB1 KD in EJ7-HEK cells. (**C**) EJ7 cNHEJ assay (shSCR vs shYB1, 95% CI = −0.46–(−0.07) p = 0.0119, shSCR vs shTP53BP1 95% CI = 0.046–0.43 p = 0.0197, one-way ANOVA, n = 3). (**D**) Western blot of YB1 KD in U2OS DR-GFP cells. (**E**) U2OS DR-GFP assay (shSCR vs shYB1 95% CI = 0.1975–0.6395 p = 0.0006, shSCR vs shCtIP 95% CI = 0.1858–0.6278 p = 0.0007, one-way ANOVA, n = 2).

To evaluate changes in canonical NHEJ- or HR-based repair, we performed distal end joining without indels assays, using the EJ7-GFP cNHEJ reporter HEK cell line, and Direct Repair HR reporter assays, using HEK293 DR-GFP and U2OS DR-GFP cells. Following double sgRNA transfection, a blunt-ended double strand break is formed with a repair mechanism specific for cNHEJ, restoring the expression of GFP (Fig. 6a)^36^. We infected EJ7-GFP cells with lentivirus containing either scramble, shYB1, or shTP53BP1 as a positive control into EJ7-GFP cells to knock down YB1 or TP53BP1 (Fig. 6b) and subsequently transfected with sgRNAs to induce double strand breaks with blunt ends. Following 72 h of recovery, YB1-depleted cells showed significantly greater percent positive GFP cells while TP53BP1-depleted cells showed fewer positive cells compared to controls (Fig. 6c). We infected HEK293-DR-GFP or U2OS DR-GFP cells with lentivirus containing either scramble, shYB1, or shCtIP as a positive control to knock down YB1 or CtIP and subsequently transfected with SCEI endonuclease plasmids to induce double strand breaks, leading to GFP restoration following HR mediated repair. Following 72 h of recovery, YB1- and CtIP-depleted U2OS-DR-GFP and HEK293-DR-GFP cells showed significantly lower levels of percent positive GFP cells compared to controls (Fig. 6d and Supp. Fig. 10). Together, these data support our hypothesis that YB1-depleted cells perform more canonical NHEJ and less HR.

### YB1 knockdown combined with radiation results in decreased proliferation and increased senescence

**Figure 7:**
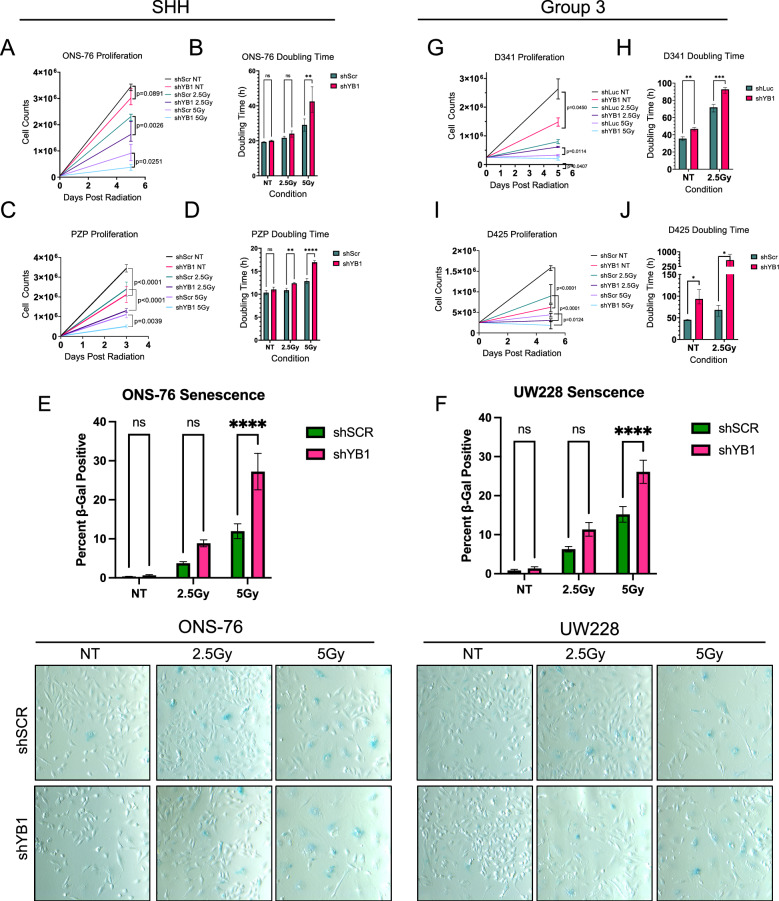
YB1 depletion results in delayed radiation response in SHH and Group 3 Medulloblastoma (**A1**) 5.0e4 ONS-76 cells irradiated at 2.5Gy and 5Gy, harvested after 4 days, and counted (3.46e6 shSCR NT vs 3.02e6 shYB1 NT p = 0.081; 2.31e6 shSCR 2.5Gy vs 1.63e6 shYB1 2.5Gy p = 0.0026; 9.10e5 shSCR 5Gy vs 3.79e5 shYB1 5Gy p = 0.0251, n = 3). (**A2**) Doubling time calculated for ONS-76 (19.4 h shSCR NT vs 20.0 h shYB1 NT p = 0.9942; 21.7 h shScr 2.5Gy vs 24.1 h p = 0.8034; 29.1 h shScr 5Gy vs 42.5 h shYB1 5Gy p = 0.0015, n = 3). (**A3**) β-Gal stain of ONS-76 cells following radiation time course demonstrating increased senescence of YB1 depleted cells compared to irradiated control (5Gy shSCR vs 5Gy shYB1 95% CI = −22.64–(−7.91) p < 0.0001 two-way ANOVA, n = 3). (**B1**) 2.5e4 Pzp53Med cells irradiated at 2.5Gy and 5Gy, harvested after 3 days, and counted (3.45e6 shSCR NT vs 2.12e6 shYB1 NT p < 0.0001; 2.42e6 shSCR 2.5Gy vs 1.31e6 shYB1 2.5Gy p < 0.0001; 1.11e5 shScr 5Gy vs 5.1e4 shYB1 5Gy p = 0.0039, n = 3). (**B2**) Doubling time calculated for Pzp53Med (10.34 h shSCR NT vs 11.04 h shYB1 NT p = 0.2149; 10.85 h shSCR 2.5Gy vs 12.37 h p = 0.0036; 12.84 h shSCR 5Gy vs 16.95 h shYB1 5Gy p < 0.0001, n = 3). (**C**) β-Gal stain of UW228 cells following radiation time course demonstrating increased senescence of YB1 depleted cells compared to irradiated control (5Gy shSCR vs 5Gy shYB1 95% CI = −16.75–(−5.09) p < 0.0001 two-way ANOVA, n = 3). (**D1**) 2.5e5 D341 cells plated and irradiated followed by a 5 day incubation period (2.6e6 shLUC NT vs 1.48e6 shYB1 NT p = 0.0114; 8.0e5 shLUC 2.5Gy vs 6.13e5 shYB1 2.5Gy p = 0.0450; 3.3e5 shLUC 5Gy vs 2.1e5 shYB1 5Gy p = 0.0407, n = 3). (**D2**) Doubling time for D341 (35.50 h shLUC NT vs 46.78 h shYB1 NT p = 0.0074; 71.83 h shLUC 2.5Gy vs 92.76 h p = 0.0001, n = 3). (**E1**) 2.5e5 D425 cells plated and irradiated followed by a 5 day incubation period (1.58E6 shSCR NT vs 6.25E5 shYB1 NT p < 0.0001; 8.97e5 shSCR 2.5Gy vs 3.02e5 shYB1 2.5Gy p < 0.0001; 4.43e5 shSCR 5Gy vs 1.85e5 shYB1 5Gy p = 0.0124, n = 3). (**E2**) Doubling time for D425 (45.2 h shSCR NT vs 93.7 h shYB1 NT p = 0.0281; 68.1 h shSCR 2.5Gy vs 575.4 h p = 0.0140, n = 3). All comparisons performed using 2-way ANOVA, see Supp Fig. 11 for growth and doubling times statistics.

Given the lack of a YB1-specific inhibitor and the challenges associated with stable knockdown in primary patient PDX models, we performed in vitro radiation time courses on several control or YB1 knockdown MB-derived cell lines and counted cells beyond 48 h of radiation (Fig. 7 and Supp. Fig. 12). In SHH group cell lines, (human ONS-76 p53 WT and mouse Pzp53Med p53 Null) the difference in cell counts between non-irradiated control and KD cells was not significant in ONS-76 but was significant in Pzp53Med cells, whereas there were clear reductions in proliferation of YB1 KD cells and significant increases in doubling time compared to control-irradiated cells for both cell lines following radiation (Fig. 7a–d). Immunoblotting of lysates collected following each timepoint shows changes in markers which corroborate the cell count data. p21, a marker of senescence and p53 activation, is increased in ONS-76 YB1 KD cells treated with 5 Gy compared to scramble controls (Supp. Fig. 11a). Following 5 Gy radiation, there was also a significant increase in β-gal-positive YB1-depleted ONS-76 cells (Fig. 7e). YB1-depleted Pzp53Med cells treated with 5 Gy had decreased CyclinD2, a marker of proliferation, compared to control cells at the same dose (Supp. Fig. 11e). However, markers of senescence (p21 and p16) were not present in these cells, likely due to the cell line being *TP53*-null. Irradiation of YB1 depleted UW228 cells resulted in a significantly higher proportion of β-gal-positive staining (Fig. 7f), an increase in p21 levels (Supp. Fig. 11d), and a decrease in colony formation capacity (Supp. Fig. 13), achieving a similar response to both ONS-76 cells. YB1 also appeared to be a driver of both proliferation and stable damage repair in Group 3 cell lines (Fig. 7g–j). Following YB1 KD in both D425 and D341 Group 3 cells, there was an increase in doubling time of non-irradiated cells which increased following radiation compared to short-hairpin control cells. For both cell lines, there was a decrease from initial plating number for the shYB1 5 Gy group, making doubling time incalculable. Immunoblotting of D425 cells showed a similar trend to SHH MB cells with a decrease in pRB, a marker of proliferation, and a peak in p21 protein levels at 24 and 48 h post-radiation (Supp. Fig. 11b,c). Taken together, these findings indicate that radiation synergizes with YB1 silencing in both SHH and Group 3 MB cells to reduce proliferation and promote senescence.

## Discussion

Here we extend our previous findings of YB1 as a driver of cell proliferation in SHH MB to include Group 3 MB and we demonstrate that YB1 promotes a more stable, HR-based mechanism of repair that is required for appropriate IR-induced damage signaling in both MB subgroups. We show that YB1 protein is robustly expressed across SHH and Group3 and 4 cell lines, it is present in patient IHC regardless of *TP53* mutational status, and overexpression results in decreased survival in the NeuroD2-SmoA1 spontaneous SHH MB mouse model. Additionally, the brain tumor stem cell compartment is thought to drive recurrence following treatment^37,38^; therefore, we mined previously published MB scRNAseq data and found that YB1 is robustly expressed in progenitor cell populations. Given the known role of YB1 in driving stemness, YB1 inhibition could lead to greater stem compartment targeting. These data indicate therapeutic significance of YB1 in MB and; therefore, a need to understand the synergy between YB1 inhibition and standard of care which includes radiation.

In the past, a role for YB1 in radiation response was inconsistent and lacked a functional outcome. Many studies have placed emphasis on YB1 proteolytic cleavage for nuclear entry and functionality^39^. However, in our SHH cells, cleavage does not appear to be required for nuclear entry or functionality, as exemplified by full length nuclear- and chromatin-fraction YB1. In addition, there is a decrease in YB1 S102 phosphorylation following radiation of MBCs (not shown), a phospho-site implicated in regulation of nuclear entry and driving DNA-binding and oncogenic phenotypes^40–42^. Thus, our data are in keeping with previous reports of YB1 being constitutively active in SHH MB through S102 phosphorylation and nuclear entry even in the absence of exogenous insult or proteolytic cleavage, while the decreased post-radiation phosphorylation is likely a result of checkpoint activation and cell cycle exit.

We show that silencing YB1 alongside radiation likely forces MB cells to use a repair method that is more rapid and potentially less amenable to mitotic catastrophe and apoptosis up to 48 h following IR, as exemplified by accelerated γH2AX reduction, enhanced repair of DNA single and double strand breaks, and a lack of multinucleation or re-accumulation of γH2AX at 24 h. However, while silencing YB1 allowed for faster recovery up to 48 h, there is an expense to viability beyond 48 h. It is also known that NHEJ can be faster than HR and that Rad51 and RPA32 S4/8 are positive markers of HR^29,43^. On the contrary, TP53BP1 and RIF1 can inhibit early repair events such as end resection through CtIP inhibition^35^. Altogether, depleting YB1 may drive the cells to utilize a more rapid and NHEJ-reliant repair pathway, resulting in genomic instability and the reduced proliferation seen 3–5 days following irradiation, which was exemplified by less RAD51 foci, reduced RPA32 phosphorylation, more TP53BP1 nuclear bodies, and more RIF1 foci in YB1-depleted cells. On the other hand, some factors may impact the accumulation and recognition of damage. In fact, damage accumulation may be contingent upon chromatin compaction, thereby affecting damage resolution. While YB1 is implicated in maintaining an open chromatin state which could make DNA more vulnerable to damage^44^, this does not appear to be the case in our studies, as all non-synchronized cells accumulated comparable levels of γH2AX and single and double strand breaks 15 min post-IR damage^45–47^.

Patients with SHH-activated and *TP53* mutant MBs have worse outcomes, likely due to the necessity of p53 for radiation and chemotherapy-induced apoptosis^48^. Even though Pzp53Med cells are p53-null and Daoy, UW228, and D425 cells are p53-mutated, all demonstrate differential repair kinetics between control and knockdown cells similar to ONS-76, MBC, and D341 cells, all of which are *TP53* WT. Indeed, depleting YB1 in Pzp53Med and D425 cells still results in lower rates of proliferation compared to control cells at 3 and 5 days following IR damage. Additionally, 24 h after IR, Daoy YB1 KD cells do not re-accumulate γH2AX compared to controls, a phenotype seen in ONS-76 and D341. While binding and inhibition of p53 by YB1 was previously reported, our data suggests p53 is not required for YB1 KD MB cell radiation response; however, whether YB1 binds and inhibits p53 in WT cells to promote survival is unclear^26,49^. Interestingly, while ONS-76 and UW228 show an increase in β-gal positive staining in irradiated YB1 depleted cells compared to control irradiated, in UW228 and D425 YB1 KD results in a substantial decrease in p16 while p21 is increased in irradiated YB1 depleted cells compared to control, which is more in line with increase in senescence. On the other hand, ONS-76 and Pzp53Med cells do not show any expression of p16 likely due to ONS-76 having low p53 expression and Pzp53Med being *TP53* null.

Finally, *MYC* amplification, a biomarker of poor outcome in Group 3 MB patients, is present in D425 and D341 cells, both of which respond favorably to YB1 KD in combination with radiation treatment. Given that cMYC was recently shown to synergize with Chk1 inhibition in a mechanism likely related to replication-driven genomic instability^50^, the proliferation decrease in non-irradiated cells following YB1 KD could result from a similar requirement for DNA repair signaling to maintain genomic stability, particularly exacerbated by radiation. Taken together, our data point to YB1 as a potential therapeutically relevant target in both SHH and Group 3 MB due to the role of YB1 in driving proliferation and in its involvement in the DNA damage response to ionizing radiation.

## Materials and methods

### IHC on human samples

All methods were carried out in accordance with Emory University’s Institutional Review Board relevant guidelines and regulations**.** De-identified patient tumor samples were provided by the Neuropathology Department of Children's Healthcare of Atlanta and studies performed on the patient tumor tissues received ethical approval by and were carried out in accordance with Emory University’s Institutional Review Board (IRB Protocol #00,045,406). All human tissues were obtained after informed consent. Immunohistochemistry on paraffin embedded and sectioned samples was performed using a standard procedure. For this, slides were deparaffinized, dehydrated and antigen retrieval was performed using Tris–EDTA Buffer, pH 9.0 (Abcam). Tissues were blocked with 5% goat serum and stained followed by DAB.

### Animal studies

All animal experimental protocols were conducted in accordance with the Emory University Institutional Animal Care and Use Committee guidelines after approval from IACUC, protocol number PROTO201700740 (AMK)**.** NeuroD2-SmoA1^51,52^ and BL6 mice were obtained from Jackson Laboratories. For survival studies 1.5e5cells/2uL of MBCs were suspended in PBS and injected into p5 BL6 pups.

### NeuroD2-SmoA1 primary cell culture

MBCs were isolated from NeuroD2-SmoA1 mouse tumors and cultured as described previously^51,52^. Cells were seeded on Matrigel (Corning) coated plates with Neurobasal medium containing penicillin/streptomycin, 1 mmol/L sodium pyruvate, 1 × B27 supplement, and 2 mmol/L L-glutamine. Primary MBCs were cultured for 4 h with 10%FBS prior to media change to No FBS at which point Lentivirus or Adenovirus were added with an incubation time of 48 h prior to experiment initiation or 24 h prior to re-implantation into BL6 mice.

### Cell lines

Mouse MB cell line Pzp53Med (p53 null, murine derived^19^) was a generous gift from Dr. Matthew Scott (Stanford). Human MB cell line D341 was obtained from ATCC. ONS-76 (p53 wildtype), Daoy (p53 Mut), and UW228 (p53 Mut) were a gift from Dr. Tobey MacDonald (Emory University), and D425 MB cell line is a gift of Dr. Eric Raabe (Johns Hopkins). For the purposes of this study Pzp53Med, ONS76, UW228, and DAOY are classified as SHH and D341 and D425 are classified as Group 3. ONS-76, UW228, and Pzp53Med cells were cultured in DMEM/F12 with 10% FBS. Daoy was cultured in EMEM supplemented with 10% FBS and D341 was cultured in EMEM supplemented with 20% FBS. D425 was cultured in DMEM supplemented with 10%FBS and Glutamine. Control shRNA constructs consisted of shscramble (shSCR), shGFP, and shLuciferase (shLUC). YBX1 knockdown in human cell lines was performed using TRCN0000315309 (referred to as shYB1_09) or TRCN0000315307 (referred to as shYB1_07) and knockdown in mouse cells was performed using TRCN0000333885 (referred to as shYB1_85) or TRCN0000077233 (referred to as shYB1_33) from Millipore Sigma. For overexpression of YB1 in primary mouse cells, replication competent adenovirus was purchased from Vector Biolabs (ADV-276442) and amplified in HEK 293 T cells prior to repeated freeze thaw lysis.

### Source of HEK293T cells

HEK293-EJ7-GFP cells were a gift of Dr. David S. Yu (Emory University) originally obtained from Jeremy Stark (City of Hope)^32^. U2OS-DR-GFP and HEK293-DR-GFP were a gift of Dr. David S. Yu (Emory University) originally obtained from Jeremy Stark (City of Hope). HEK293T packaging cells were a gift of Dr. Shubin Shahab obtained originally from ATCC (American Type Culture Collection).

### Western blotting

Tissues or cells were homogenized and lysed in RIPA lysis buffer with protease inhibitor cocktail (CST 5871), and phosphatase inhibitors. A total of 15–30 μg of each sample was denatured and separated on 14% SDS-PAGE gels, then transferred to immobilon-PVDF membranes (Millipore). For quantification purposes, chemiluminescent signals of post-translational modifications were normalized to total protein prior to normalization to β-Tubulin. For blots of the same molecular weight, two blots were run with the same samples without stripping of blots. All blots were trimmed at the specified molecular weights prior to probing with primary antibodies based on sizing determined from previous literature. Blots were imaged using ECL and X-ray film. Representative blot cutting patterns and representative films are available in the supplementary file (Immuno-blotting supplementary Figs. 1–16). For western blotting of irradiated samples, all samples within one blot were X-rayed simultaneously followed by time course harvesting. The following antibodies were used: YB1 (D299 CST), γH2AX (immunofluorescence: MA1-2022 Thermo Fisher, Immunoblotting/Immunofluorescence: D7T2V CST), H2AX (D17A3 CST), CyclinD1 (55506 CST), Chk1(2G1D5 CST), pChk1 Ser317 (D12H3 CST), Chk2 (D9C6 CST), pChk2 Thr68 (C13C1 CST), β-Tubulin (sc-166729 SCBT), α-Tubulin (#2144 CST), GAPDH (D16H11 CST), p21 (12D1 CST), LaminB1 (D4Q4Z CST), pRb Ser780 (D59B7 CST), Rb (D20 CST), TP53BP1 (A300-273A Bethyl Labs), Rad51 (PC130 Calbiochem), CyclinA (611268 BD Biosciences), RIF1 (A300-569A Bethyl), LaminA/C (4777 CST). Acronyms: CST—Cell Signaling Technology, SCBT—Santa Cruz Biotechnology.

### Radiation dosing

Cell models were irradiated in a cell line dose-dependent manner chosen based on evaluation of IR phenotypic effects up to 48 h and beyond 48 h. ONS-76 and Daoy were treated with 10 Gy for time courses leading up to 48 h or 2.5 Gy or 5 Gy for time points beyond 48 h. PZP, D341, and D425 were treated with 2.5 Gy and 5 Gy for both experiments leading up to 48 h and beyond 48 h. MBCs were treated with 2 Gy for all experiments.

### EJ7 NHEJ and DR-GFP assay

Distal EJ without indels assay in HEK293 EJ7 line: HEK293 EEJ7 cells were infected with either shScr or shYB1 lentivirus. Twenty-four hours later, media was removed, and cells were transfected with 3 ug I-SceI and 2 ug of both sgRNA7a and sgRNA7b plasmids. Forty-eight hours later, cells were harvested, washed twice with PBS, resuspended in PBS and subjected to flow cytometry (Aurora Cytek) for GFP fluorescence. To measure cNHEJ efficiency, the percentage of GFP positive cells (c-NHEJ positive) was analyzed using the FlowJo software**.** U20S DR-GFP or HEK293 DR-GFP cells were plated in 10 cm dishes and the next day transfected with the shRNA of interest. After twenty-four hours, cells were transfected with 5 ug I-SceI plasmid. Seventy-two hours later, cells were harvested, washed twice with PBS, resuspended in PBS and subjected to flow cytometry (Aurora Cytek) for GFP. To measure HR efficiency, percentage of GFP positive cells (HR positive) was analyzed using the FlowJo software. For DR-GFP assay biological replicates are presented separately with three technical replicates each.

### Cell synchronization experiments

Cells were seeded into 10 cm dishes or 24 well dishes for 24 h prior to addition of Aphidicolin (Final concentration 10ug/mL) with an incubation time of 24 h. Media was replaced 10 min prior to irradiation.

### Flow cytometry

Cell cycle analysis was performed using the Click-iT™ EdU Alexa Fluor™ 647 Flow Cytometry Assay Kit (ThermoFisher) according to the manufacturer’s specifications. Briefly, cells were incubated with 10 uM EdU for 1 h prior to trypsinization and staining with LIVE/DEAD™ Fixable Aqua Stain (ThermoFisher). Cells were washed twice with PBS and then fixed and permeabilized using the BD Cytofix/Cytoperm™ (BD Biosciences) kit. Cells were then stained using the ThermoFisher Alexa Fluor™ 647 EdU azide and Propidium Iodide (Biolegend). Samples were analyzed on a Beckman Coulter CytoFLEX flow cytometer. All flow analysis was performed using FlowJo (TreeStar). Doublets were gated out for primary analysis and included as separate information.

### Immunofluorescence and analysis

Cells were fixed for 10 min in fresh 4% Formaldehyde (made fresh from PFA) prior to 3 × wash with PBS. Cells were permeabilized with 0.3% Triton X-100 and blocked with 0.5% Bovine Serum Albumin and 3% Normal Goat Serum. Primary Antibody was added overnight at 4C and secondary was added for 1 h at RT. Cell imaging was performed on the Olympus FV1000 at the Emory University Integrated Cellular Imaging Core. Quantification analysis for all foci was performed using CellProfiler software with one set of parameters for all images specific to each image set. A minimum of three images were acquired per condition for all images presented throughout the paper. For biological replicates data was combined prior to statistical analysis.

### Comet assay

Cells were seeded into a 24 well plate 24 h prior to irradiation. Cells were then trypsinized, inactivated with media containing 10% FBS, centrifuged, and resuspended in 0.5% low melt agar prior to aliquoting onto comet slides. Electrophoresis was performed according to the Trevigen Comet Assay kit (cat# 4250-050-K). A minimum of three images were acquired per condition per biological replicate. All conditional pairs (NT, 15 min, 6 h) were imaged using the same acquisition parameters (Gain and Exposure time) prior to quantification using Open Comet plugin for ImageJ or Cell Profiler.

### β-galactosidase staining

Cells were seeded into a 6 well plate 48 h prior to radiation. 96 h following radiation cells were fixed and stained for β-Galactose using the Cell Signal Technology kit (9860) followed by cell staining with SYBR Gold and overlay with Glycerol. Three images were taken per well with percentages calculated as β-Gal positive cells over total SYBR Gold positive cells.

### Data availability statement (single cell sequencing)

Single cell sequencing data was obtained from the UCSC cell browser https://d33sxa6bpqwi51.cloudfront.net/ by searching expression data for *YBX1*.

### Statistical analysis

Statistical comparisons were performed using GraphPad Prism. 2-way ANOVA were used for Flow Experiments, cell count, and doubling time comparisons. 1-way ANOVA was used for β-Galactosidase comparisons. For non-gaussian datasets, including comet assays and foci counts, Kruskal–Wallis test is used. For densitometry, ratio-paired t test was used. Bar graphs plot SEM and box and whisker plots contain data range with Tukey plots where applicable.

This study is conducted in accordance with ARRIVE guidelines.

## Supplementary Information

Supplementary Information.

## Data Availability

The authors confirm that the data supporting the findings of this study are available within the article [and/or] its supplementary materials.
