# Author Correction: YB1 modulates the DNA damage response in medulloblastoma

**DOI:** 10.1038/s41598-023-36594-3

**Published:** 2023-06-15

**Authors:** Leon F. McSwain, Claire E. Pillsbury, Ramona Haji‑Seyed‑Javadi, Sandip Kumar Rath, Victor Chen, Tiffany Huang, Shubin W. Shahab, Haritha Kunhiraman, James Ross, Gabrielle A. Price, Abhinav Dey, Dolores Hambardzumyan, Tobey MacDonald, David S. Yu, Christopher C. Porter, Anna M. Kenney

**Affiliations:** 1 Department of Pediatrics, Emory University, 1760 Haygood Dr., Atlanta, GA 30322 USA; 2 Winship Cancer Institute, Emory University, Atlanta, GA 30322 USA; 3 Department of Biology, Emory University, Atlanta, GA 30322 USA; 4 Department of Microbiology and Immunology, Emory Vaccine Center, Emory University, Atlanta, GA USA; 5 Department of Neurosurgery, Icahn School of Medicine at Mount Sinai, New York, NY 10029 USA

Correction to: *Scientific Reports* https://doi.org/10.1038/s41598-023-35220-6, published online 19 May 2023

The original version of this Article contained errors in the legends of Figures 1, 2, 3, 4, 5, 6 and 7.

In Figure legend 1,

“YB1 is expressed across all MB subgroups and overexpression is associated with shortened survival in an SHH primary mouse model.”

now reads:

“YB1 is expressed across all MB subgroups and overexpression is associated with shortened survival in an SHH primary mouse model (**A**) Immunoblotting cell lysate from SHH cells [MBC (primary NestinD2-SmoA1), Pzp53Med (Ptch-LacZ-p53null), Daoy, UW228, and ONS-76], and group 3/4 cells (D341, D556, BT52, D283, and CHLA01) with GAPDH as control. (**B**) Immunohistochemistry of SHH subgroup samples from both TP53 wild type and mutated patients showing positive staining of both Nestin (Stem marker) and YB1 (Left scale bar = 100 μm, quantification Supp Fig. 1a and b). (**C**) Survival analysis of BL6 mice orthotopically implanted with NestinD2-SmoA1 primary cells following adenoviral overexpression of YB1 (GFP median survival 26.5 days YB1 median survival 60.5 days *p* < 0.0001). (**D**) UMAP of previously published single cell sequencing analysis of SHH-Math-Cre-SmoM2 showing enrichment of YB1 in numerous cell populations collected from UCSC Cell Browser: active cell cycling (MS-A1 and MS-A2) and progenitor (MS-B1). Expression profile is subdivided into 10 expression ranges apart from no expression and percent of all cells within each range listed on right.”

In Figure legend 2,

“YB1 depletion results in differential cell cycling and reduction of aberrant nuclear morphology following radiation.”

now reads:

“YB1 Knockdown results in differential cell cycling and reduction of aberrant nuclear morphology following radiation (**A**) ONS-76 shGFP and shYB1 cells were untreated (NT) or treated with 10 Gy and analyzed for cell cycle phase proportions (sub-G1, G0/G1, S, and G2/M) at 24 (shown) and 48 h using EdU and PI. Quantification of cell cycle phase distribution (SD and Means Supp Fig. 2, n = 3). (**B**) At 48 h post-irradiation, shYB1 shows significantly lower proportions of cells in sub-G1 (shGFP vs. shYB1 95% CI 0.36–6.47 *p* = 0.0275, n = 3) (**C**) and cells appearing in doublets (shGFP vs. shYB1 95% CI 3.303–14.56 *p* = 0.0026, n = 3) (**D**) (doublets excluded from cell cycle analysis). (**E**) shSCR and shYB1 non-treated and treated with 10 Gy were stained with LaminA/C and DAPI 48 h after 10 Gy irradiation. shSCR cells demonstrate more aberrations in nuclear morphology (UW228 and additional images Supp Fig. 3).”

In Figure legend 3,

“YB1 depletion results in differential γH2AX resolution and CHK2 phosphorylation in SHH and Group 3 medulloblastoma cells.”

now reads:

“YB1 depletion results in differential γH2AX resolution and Chk2 phosphorylation in SHH and Group3 medulloblastoma cells (**A**,**B**) MBCs plated for 24 h prior to infection with either control or YB1 overexpressing adenovirus. Following 48 h incubation cells were irradiated with 2 Gy and either lysed (immunoblotting) or fixed prior to staining (immunofluorescence). (**C**) D341 shLuc (control) and shYB1 cells irradiated with 5 Gy (Expanded western Supp Fig. 5). (**D**) ONS-76 shGFP (control) and shYB1 cells irradiated with 10 Gy (Additional replicates Supp Fig. 5).”

In Figure legend 4,

“YB1 depletion results in accelerated repair, γH2AX resolution, and lack of RPA32 phosphorylation at Serines 4/8.”

now reads:

“YB1 depletion results in accelerated physical repair, γH2AX resolution, and lack of RPA32 phosphorylation at Serines 4/8 (**A1**,**A2**) Neutral comet assay tail moment of ONS-76 shGFP and shYB1 treated with 10 Gy showing non-significant differences in damage accumulation (shGFP vs. shYB1, shGFP 95% CI 171–164.6 shYB1 95% CI 135–178.3 *p* > 0.9999, Kruskal–Wallis test, n = 3) and significant differences in damage resolution at 6 h (shGFP vs. shYB1, shGFP 95% CI 62.86–86.59 shYB1 95% CI 19.06–30.49 *p* < 0.0001, Kruskal–Wallis test, n = 3). (**B1**,**B2**) Alkaline comet assay tail moment of ONS-76 shGFP and shYB1 treated with 10 Gy showing nonsignificant differences in damage accumulation (shGFP vs. shYB1, shGFP 95% CI 180.5–201.5 shYB1 95% CI 183.3–207.0 *p* > 0.9999, Kruskal–Wallis test, n = 3) and significant differences in damage resolution at 6 h (shGFP vs. shYB1, shGFP 95% CI 80.1–105.1 shYB1 95% CI 37.84–57.05 *p* < 0.0001, Kruskal–Wallis test, n = 3). (**C**) Synchronization of ONS-76 with Aphidicolin for 24 h prior to radiation time course at 10 Gy (Additional replicates Supp Fig. 6). (**D**) Densitometry of pRPA32 S4/8 shows consistent elevation in shGFP cells compared to shYB1 6 h post-IR (shGFP vs. shYB1 95% CI − 0.24–0.56 *p* = 0.0223, Ratio paired t-test, n = 3, internal normalization to shGFP 6 h).”

In Figure legend 5,

“YB1 depleted cells accumulate less RAD51 foci and more TP53BP1 nuclear bodies during and after S-Phase repair.”

now reads:

“YB1 depleted cells accumulate less RAD51 and more TP53bp1 foci during and after S-Phase repair. (**A**,**B**) Aphidicolin S-phase synchronization of shGFP and shYB1 ONS-76 24 h prior to radiation at 10 Gy results in greater TP53BP1 accumulation in shYB1 cells that is sustained until 6 h and reappears at 24 h (2 h Mean rank diff. = − 149.0 *p* = 0.0045, 4 h mean rank diff. = − 168.9 *p* < 0.0004, 6 h Mean rank diff. = 35.54 *p* > 0.9999, 24 h mean rank diff. = − 208.7 *p* < 0.0001, Kruskal–Wallis test, n = 3). (**C** and Supp Fig. 8a) Non-Synchronized ONS-76 exposed to 10 Gy results in greater TP53BP1 accumulation in shYB1 cells that is sustained until 24 h (2 h Mean rank diff. = − 121.0 *p* = 0.0080, 4 h Mean rank diff. = 52.42 *p* = 0.7142, 6 h Mean rank diff. = − 117.1 *p* = 0.0117, 24 h Mean rank diff. = − 34.30 *p* > 0.9999, Kruskal–Wallis test, n = 2). (**D**,**E**) Aphidicolin S-phase synchronization of shGFP and shYB1 ONS-76 24 h prior to radiation at 10 Gy results in reduced RAD51 accumulation in shYB1 cells up to 6 h and at 24 h (4 h Mean rank diff. = 141.0 *p* = 0.0018, 6 h Mean rank diff. = 234.9 *p* < 0.0001, 24 h Mean rank diff. = 135.6 *p* = 0.0087, Kruskal–Wallis test, n = 3). (**F** and Supp Fig. 8b) Non-Synchronized ONS-76 exposed to 10 Gy results in reduced RAD51 accumulation in shYB1 cells up to 6 h and at 24 h (4 h Mean rank diff. = 41.01 *p* = 0.8115, 6 h Mean rank diff. = 144.3 *p* = 0.0007, 24 h Mean rank diff. = 176.9 *p* = 0.0031, Kruskal–Wallis test, n = 2). (**D**,**G**) Aphidicolin S-phase synchronization of shGFP and shYB1 ONS-76 24 h prior to radiation at 10 Gy results in faster γH2AX resolution up to 6 h that reappears in shGFP control at 24 h (NT Mean rank diff. = 14.48 *p* > 0.9999, 2 h Mean rank diff. = 82.26 *p* = 0.2664, 4 h Mean rank diff. = 406.7 *p* < 0.0001, 6 h Mean rank diff. = 245.9 *p* < 0.0001, 24 h Mean rank diff. = 125.3 *p* = 0.0205, Kruskal–Wallis test, n = 3).”

In Figure legend 6,

“YB1 depletion results in greater canonical NHEJ and lower HR.”

now reads:

“YB1 depletion results in greater canonical NHEJ and lower HR (**A**) Schematic of distal EJ without indels assay whereby two sgRNAs are co-transfected with SCEI to generate blunt ends repairable through cNHEJ to restore GFP expression. (**B**) Western blot of YB1 KD in EJ7-HEK cells. (**C**) EJ7 cNHEJ assay (shSCR vs. shYB1, 95% CI − 0.46–(− 0.07) *p* = 0.0119, shSCR vs. shTP53BP1 95% CI 0.046–0.43 *p* = 0.0197, one-way ANOVA, n = 3). (**D**) Western blot of YB1 KD in U2OS DR-GFP cells. (**E**) U2OS DR-GFP assay (shSCR vs. shYB1 95% CI 0.1975–0.6395 *p* = 0.0006, shSCR vs. shCtIP 95% CI 0.1858–0.6278 *p* = 0.0007, one-way ANOVA, n = 2).”

In Figure legend 7,

“YB1knockdown combined with radiation results in decreased proliferation and increased senescence in SHH and Group 3 cells.”

now reads:

“YB1 depletion results in delayed radiation response in SHH and Group 3 Medulloblastoma (**A1**) 5.0e4 ONS-76 cells irradiated at 2.5 Gy and 5 Gy, harvested after 4 days, and counted (3.46e6 shSCR NT vs. 3.02e6 shYB1 NT *p* = 0.081; 2.31e6 shSCR 2.5 Gy vs. 1.63e6 shYB1 2.5 Gy *p* = 0.0026; 9.10e5 shSCR 5 Gy vs. 3.79e5 shYB1 5 Gy *p* = 0.0251, n = 3). (**A2**) Doubling time calculated for ONS-76 (19.4 h shSCR NT vs. 20.0 h shYB1 NT *p* = 0.9942; 21.7 h shScr 2.5 Gy vs. 24.1 h *p* = 0.8034; 29.1 h shScr 5 Gy vs. 42.5 h shYB1 5 Gy *p* = 0.0015, n = 3). (**A3**) β-Gal stain of ONS-76 cells following radiation time course demonstrating increased senescence of YB1 depleted cells compared to irradiated control (5 Gy shSCR vs. 5 Gy shYB1 95% CI − 22.64–(− 7.91) *p* < 0.0001 two-way ANOVA, n = 3). (**B1**) 2.5e4 Pzp53Med cells irradiated at 2.5 Gy and 5 Gy, harvested after 3 days, and counted (3.45e6 shSCR NT vs. 2.12e6 shYB1 NT *p* < 0.0001; 2.42e6 shSCR 2.5 Gy vs. 1.31e6 shYB1 2.5 Gy *p* < 0.0001; 1.11e5 shScr 5 Gy vs. 5.1e4 shYB1 5 Gy *p* = 0.0039, n = 3). (**B2**) Doubling time calculated for Pzp53Med (10.34 h shSCR NT vs. 11.04 h shYB1 NT *p* = 0.2149; 10.85 h shSCR 2.5 Gy vs. 12.37 h *p* = 0.0036; 12.84 h shSCR 5 Gy vs. 16.95 h shYB1 5 Gy *p* < 0.0001, n = 3). (**C**) β-Gal stain of UW228 cells following radiation time course demonstrating increased senescence of YB1 depleted cells compared to irradiated control (5 Gy shSCR vs. 5 Gy shYB1 95% CI − 16.75–(− 5.09) *p* < 0.0001 two-way ANOVA, n = 3). (**D1**) 2.5e5 D341 cells plated and irradiated followed by a 5 day incubation period (2.6e6 shLUC NT vs. 1.48e6 shYB1 NT *p* = 0.0114; 8.0e5 shLUC 2.5 Gy vs. 6.13e5 shYB1 2.5 Gy *p* = 0.0450; 3.3e5 shLUC 5 Gy vs. 2.1e5 shYB1 5 Gy *p* = 0.0407, n = 3). (**D2**) Doubling time for D341 (35.50 h shLUC NT vs. 46.78 h shYB1 NT *p* = 0.0074; 71.83 h shLUC 2.5 Gy vs. 92.76 h *p* = 0.0001, n = 3). (**E1**) 2.5e5 D425 cells plated and irradiated followed by a 5 day incubation period (1.58E6 shSCR NT vs. 6.25E5 shYB1 NT *p* < 0.0001; 8.97e5 shSCR 2.5 Gy vs. 3.02e5 shYB1 2.5 Gy *p* < 0.0001; 4.43e5 shSCR 5 Gy vs. 1.85e5 shYB1 5 Gy *p* = 0.0124, n = 3). (**E2**) Doubling time for D425 (45.2 h shSCR NT vs. 93.7 h shYB1 NT *p* = 0.0281; 68.1 h shSCR 2.5 Gy vs. 575.4 h *p* = 0.0140, n = 3). All comparisons performed using 2-way ANOVA, see Supp Fig. 11 for growth and doubling times statistics.”

The original Article has been corrected.

