# Supplementary material for: YB1 modulates the DNA damage response in medulloblastoma
Source: Sci Rep. 2023 May 19;13:8087. doi: 10.1038/s41598-023-35220-6 (PMC10199100; doi:10.1038/s41598-023-35220-6)
Supplement: Supplementary file 1 — Supplementary Information. [file 41598_2023_35220_MOESM1_ESM.pdf]

YB1 Modulates the DNA Damage Response in Medulloblastoma

Leon F McSwain[1], Claire E Pillsbury[1], Ramona Haji-Seyed-Javadi[3], Sandip Kumar Rath[3], Victor Chen[2], Tiffany Huang[2], Shubin W. Shahab[1], Haritha Kunhiraman[1], James Ross[4], Gabrielle A. Price[5], Abhinav Dey[1], Dolores Hambardzumyan[5], Tobey MacDonald[1,3], David S. Yu,[3] Christopher C. Porter[1,3], Anna M. Kenney[1,3]

1. Department of Pediatrics, Emory University, Atlanta, GA, 30322
2. Department of Biology, Emory University, Atlanta, GA, 30322
3. Winship Cancer Institute, Emory University, Atlanta, GA, 30322
4. Department of Microbiology and Immunology, Emory Vaccine Center, Emory University, Atlanta, Georgia
5. Department of Neurosurgery, Icahn School of Medicine at Mount Sinai, New York, New York 10029

Running Title: **YB1 and Medulloblastoma DNA Repair**

Keywords: Radiation, YBX1, Medulloblastoma, Sonic Hedgehog, Group 3

Financial Support: This work was supported by NINDS R01NS110386 (AMK), NCI Winship Cancer Institute P30 Center Grant CA138292 (AMK, TJD, CCP), Alex's Lemonade Stand Foundation, and CURE Childhood Cancer Foundation (CCP).

Corresponding Author: Dr. Anna Marie Kenney Anna.Kenney@emory.edu 1760 Haygood Dr. Atlanta GA 30322

The authors declare no conflicts of interest

Word Count: 7110, Figure Number: 7, Supplementary Figure Number: 13

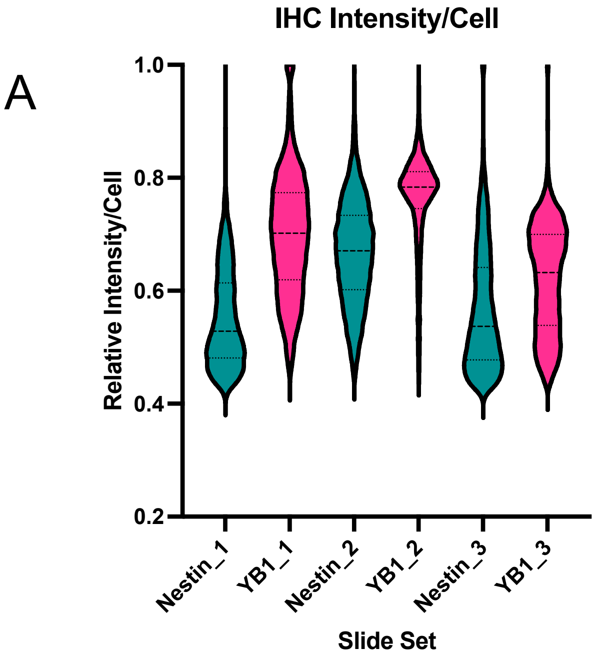

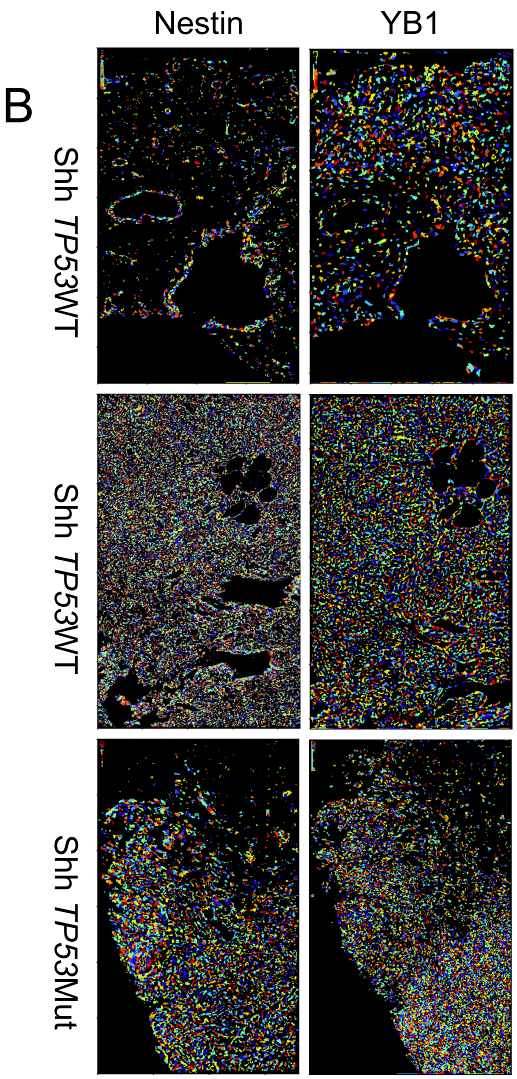

Supplementary Figure 1: (A) Quantification of cell intensity in MB patient IHC samples (Figure 1) using Cell Profiler. Cells above chosen threshold (0.4) are shown in multicolor **(B**). (**C)** Previously published single cell sequencing of MYC-driven p53 dominant negative Group 3 spontaneous mouse model (GP3-Myc-dnP53) with cell populations corresponding to active cell cycling active cell cycling (MP-A1, -2), progenitor (MP-B1,-B2, -B3), and differentiated neoplastic cell populations (MP-C1, -C2).

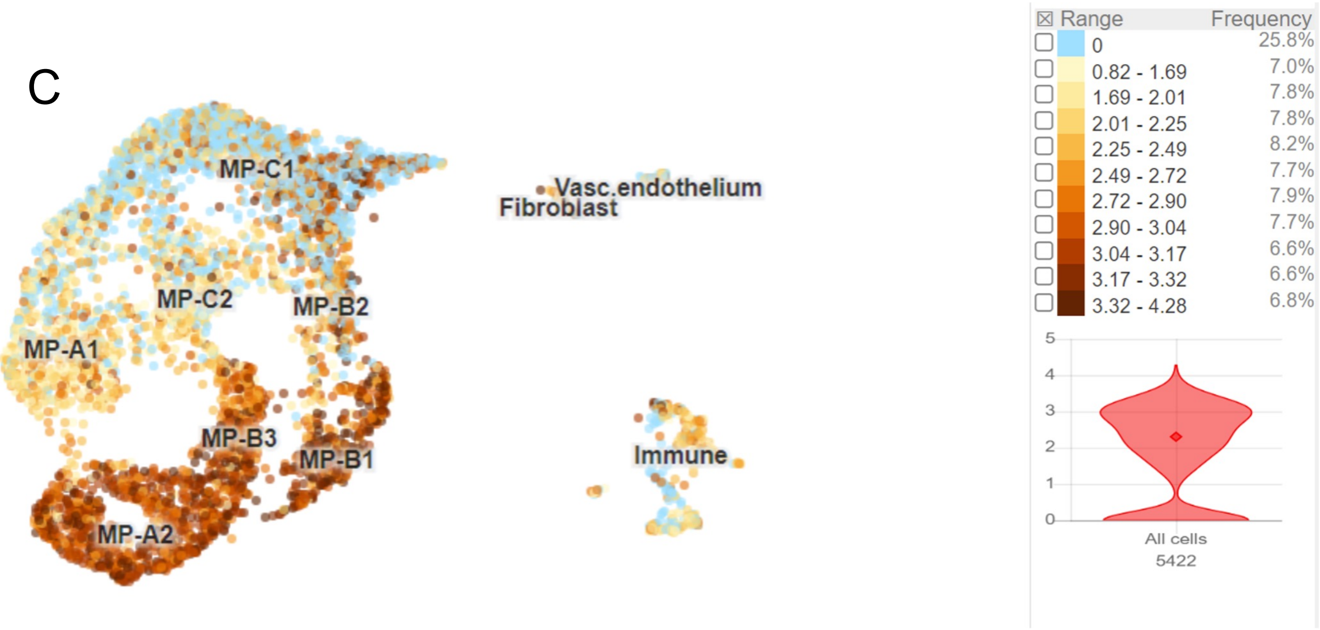

Condition	Sub G1	G0/G1	S	G2/M
shGFP NT	1.46	78.10	9.86	9.87
shYB-1 NT	0.80	77.53	6.01	13.47
shGFP 10 Gy (24 Hours)	1.81	85.33	1.11	9.88
shYB-1 10 Gy (24 Hours)	1.12	80.90	1.39	14.90
shGFP 10 Gy (48 Hours)	4.79	81.07	1.11	11.72
shYB-1 10 Gy (48 Hours)	1.37	81.60	1.87	13.63

Condition	Sub G1	G0/G1	S	G2/M
shGFP NT	0.70	0.89	3.70	2.63
shYB-1 NT	0.47	3.00	3.63	1.70
shGFP 10 Gy (24 Hours)	1.09	10.57	0.61	7.70
shYB-1 10 Gy (24 Hours)	0.79	0.82	0.62	2.14
shGFP 10 Gy (48 Hours)	2.65	6.55	0.78	3.16
shYB-1 10 Gy (48 Hours)	1.20	3.72	1.68	2.83

Comparison	Sub-G1	Doublets
NT	-2.400 to 3.713	-5.030 to 6.230
10 Gy (24 Hours)	-2.366 to 3.746	-2.764 to 8.497
10 Gy (48 Hours)	0.3602 to 6.473	3.303 to 14.56

Supplementary Figure 2: Percent averages across three independent experiments of ONS-76 cell cycle analysis for shGFP and shYB1 cells non-irradiated and irradiated at 10Gy (**Figure 2**). Cell cycle phase on x axis and cell condition on y axis. Mean of cell cycle (**Top**), SD of cell cycle (**Middle**). (**Bottom**) 95% CI for comparisons in Figure 2 C and D.

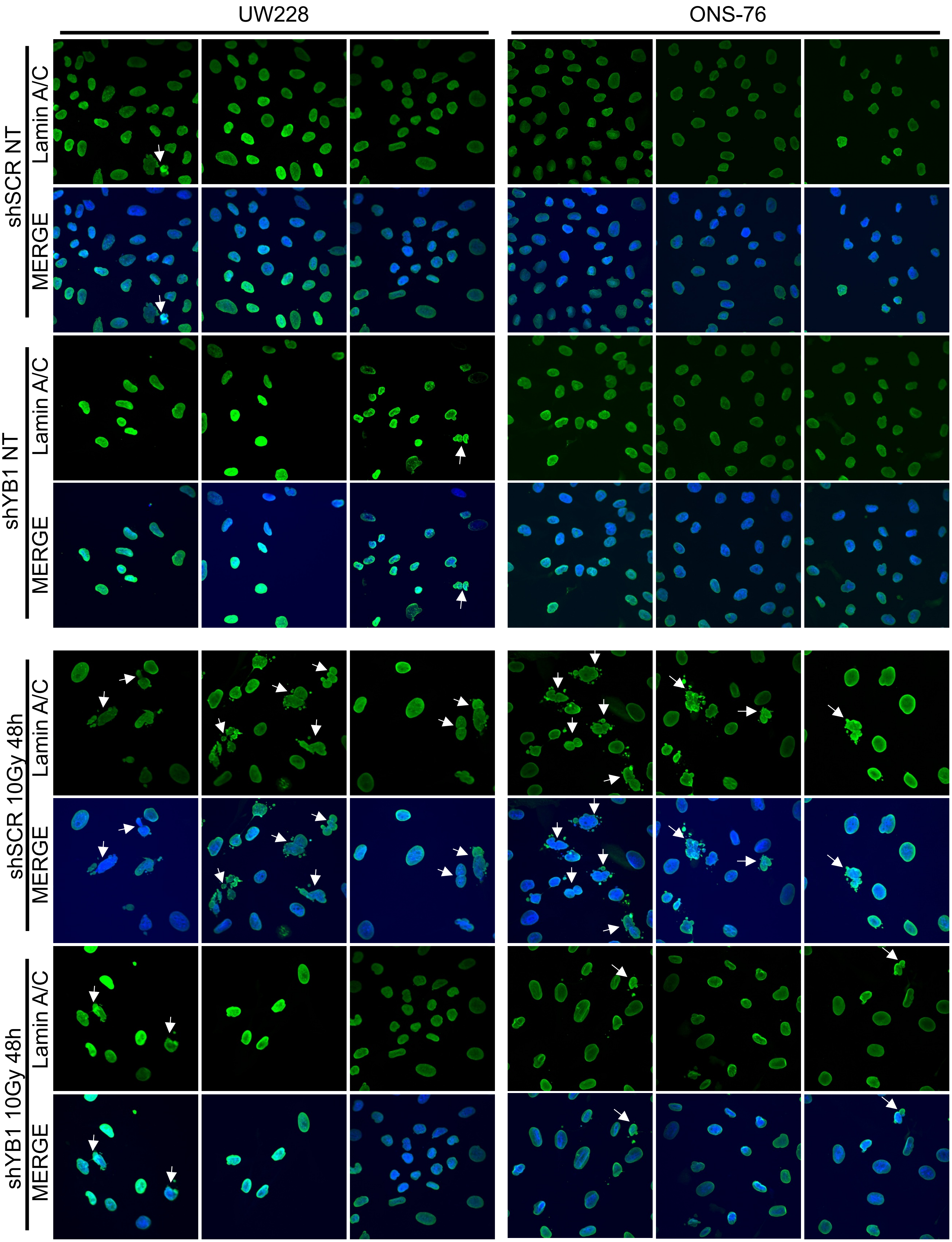
UW228
ONS-76
shSCR NT
Lamin A/C
MERGE
shYB1 NT
Lamin A/C
MERGE
shSCR 10Gy 48h
Lamin A/C
MERGE
shYB1 10Gy 48h
Lamin A/C
MERGE

Supplementary Figure 3: Representative images of nuclear envelope (stained with LaminA/C) in ONS-76 p53wt (**Right**) and UW228 p53Mut (**Left**) following 10Gy irradiation. Following 48h, there is increased aberrations in nuclear morphology in shSCR cells compared to shYB1.

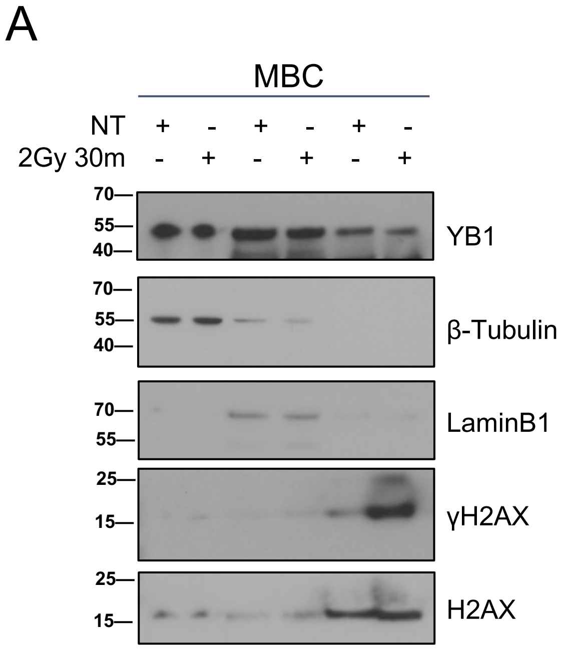

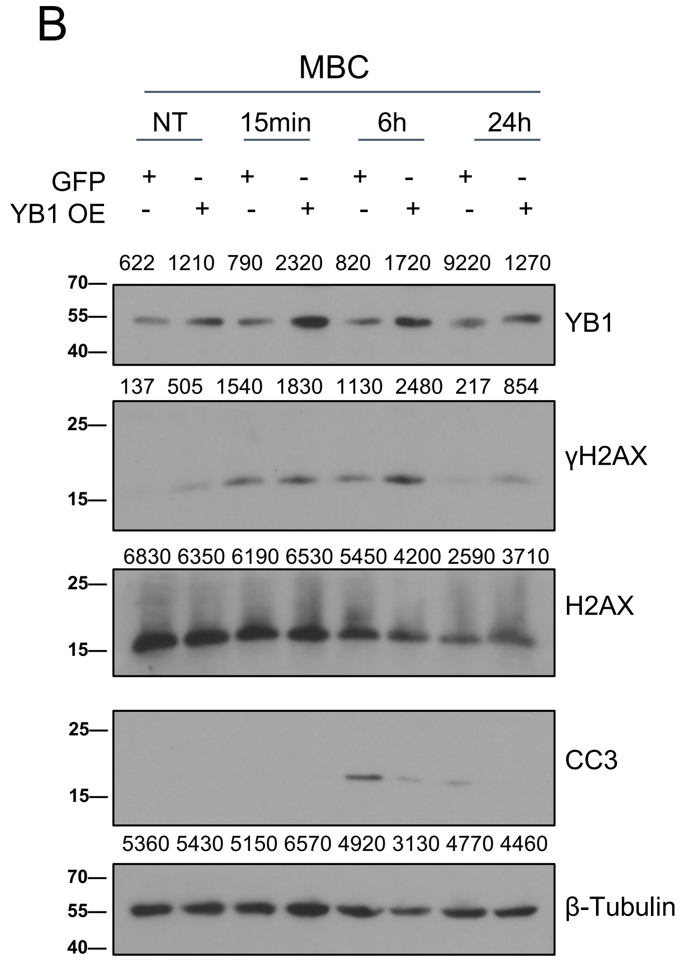

	GFP NT	YB1 NT	GFP 15m	YB1 15m	GFP 6h	YB1 6h	GFP 24h	YB1 24h
γH2AX/H2AX	0.000	0.000	0.229	0.201	0.187	0.511	0.064	0.151
γH2XA/H2AX/βtub	0.000	0.000	0.444	0.306	0.381	1.632	0.134	0.338
CC3/βtub	0.000	0.000	0.000	0.000	0.354	0.087	0.092	0.000

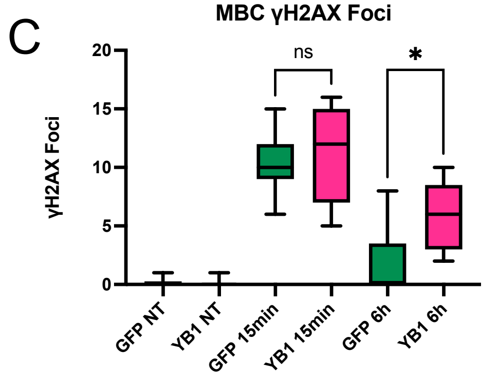

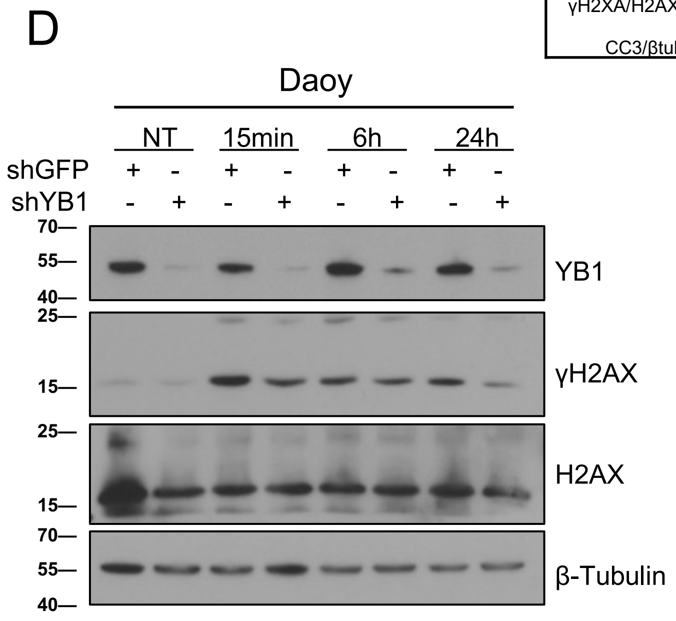

Supplementary Figure 4: (A) Primary MBCs, non-treated and treated with 2Gy 30min following 24h of plating, fractionated into cytoplasmic, nuclear, and chromatin. β-Tubulin (Cytoplasm), LaminB1 (Nucleus), and H2AX (Chromatin), serve as the fraction controls. **(B)** Densitometry of MBC YB1 OE time course at 2Gy (Figure 3) with either normalization to H2AX or H2AX and β-tubulin. NT density was subtracted from each condition within group. **(C)** Representative IF of MBCs irradiated with 2Gy show similar levels of γH2AX foci accumulation at 15min but a difference in resolution at 6h (GFP NT SEM = 0.11, YB1 NT SEM = 0.083, GFP 15min SEM = 0.7519, YB1 15min SEM = 1.16, GFP 6h SEM = 0.72, YB1 6h SEM = 0.78, GFP 6h vs YB1 6h p=0.0354 mean rank diff. = -19.38, Kruskal-Wallis test, n=1). **(D)** Daoy cell time course western blot of YB1 KD cells showing persistence of γH2AX in shGFP at 24 hours following 10Gy radiation.

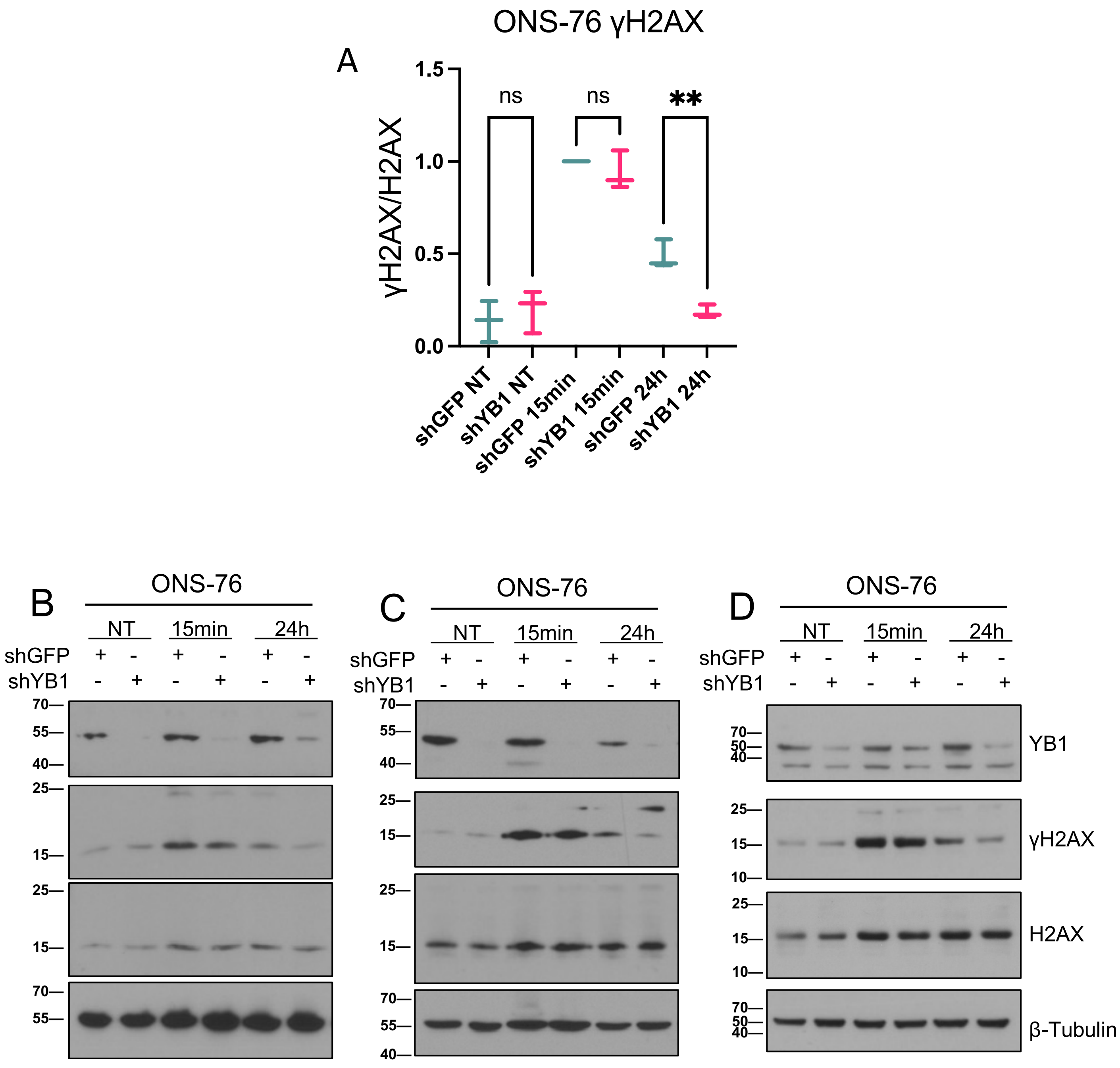

Supplementary figure 5: (A) Densitometry of three biological replicates of ONS-76 time courses for cells treated at 10Gy **(B-D)** normalized to shGFP 15min (shGFP vs shYB1 24h 95% CI = 0.1086-0.4971 p=0.0030, Ordinary one-way ANOVA, n=3)**.**

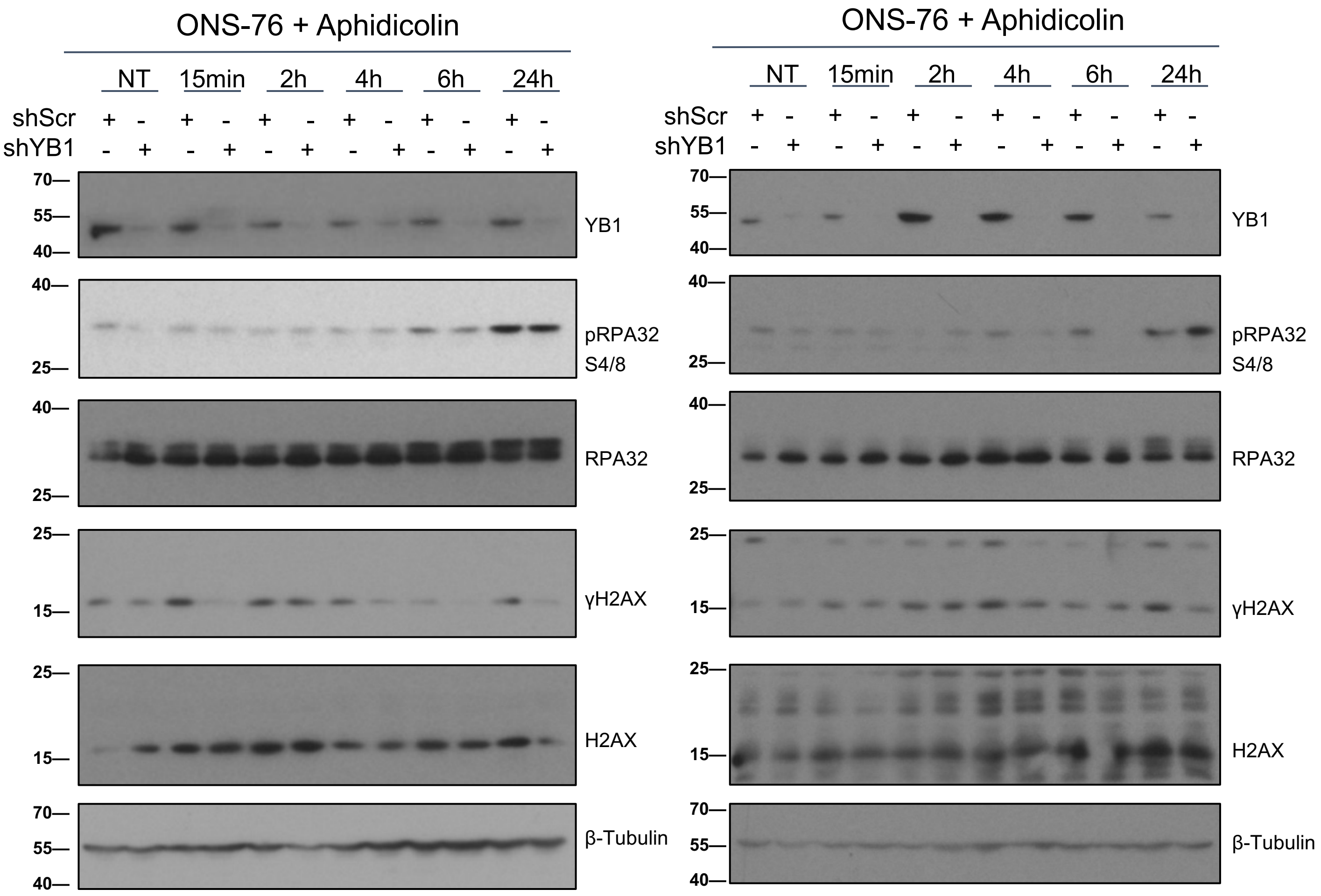

Supplementary Figure 6: (**B and C**) biological replicates of ONS-76 Aphidicolin experiments for RPA32 Densitometry (Figure 4)

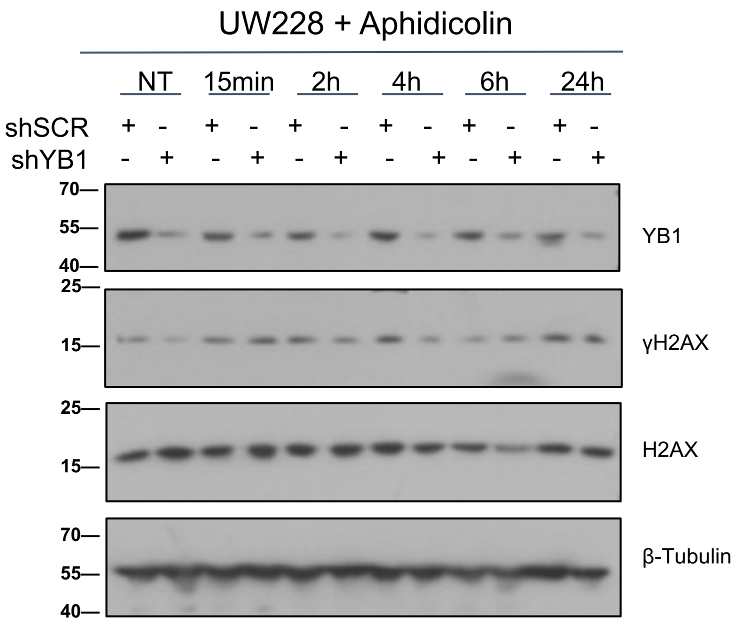

Supplementary Figure 7: 10Gy radiation of UW228 shSCR and shYB1 cells following S-phase synchronization with aphidicolin.

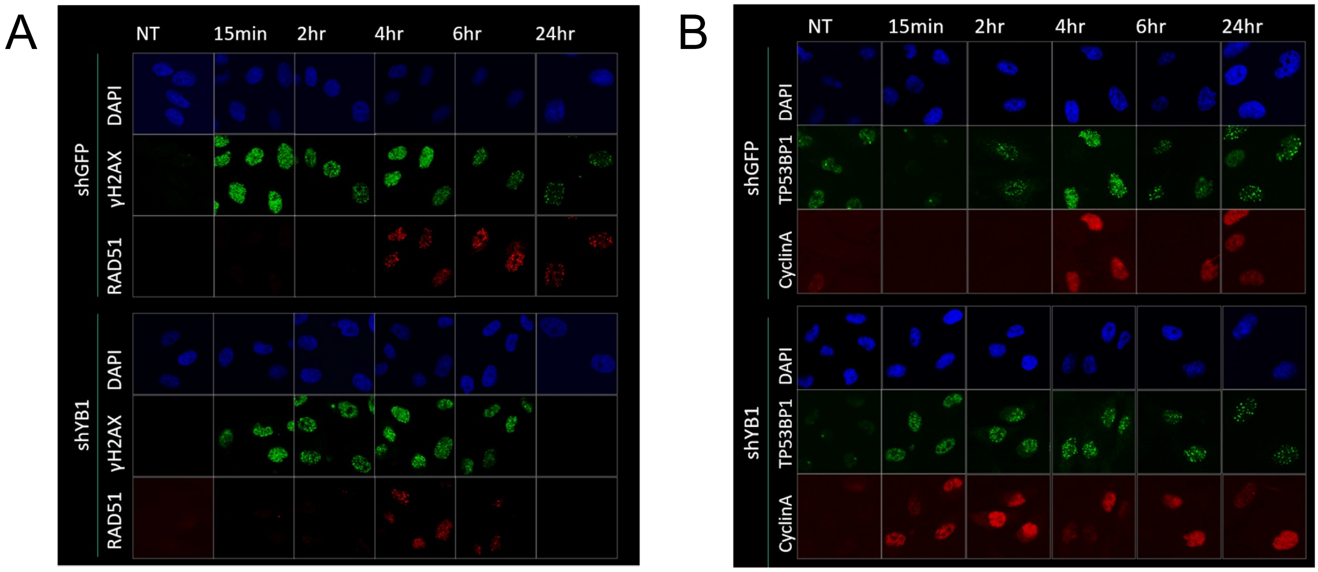

Supplementary Figure 8: (A) Non-Synchronized ONS-76 exposed to 10Gy results in greater TP53BP1 accumulation in shYB1 cells that is sustained until 24h (stats in main paper). **(B)** Non-Synchronized ONS-76 exposed to 10Gy results in reduced RAD51 accumulation in shYB1 cells up to 6h and at 24h (stats in main paper).

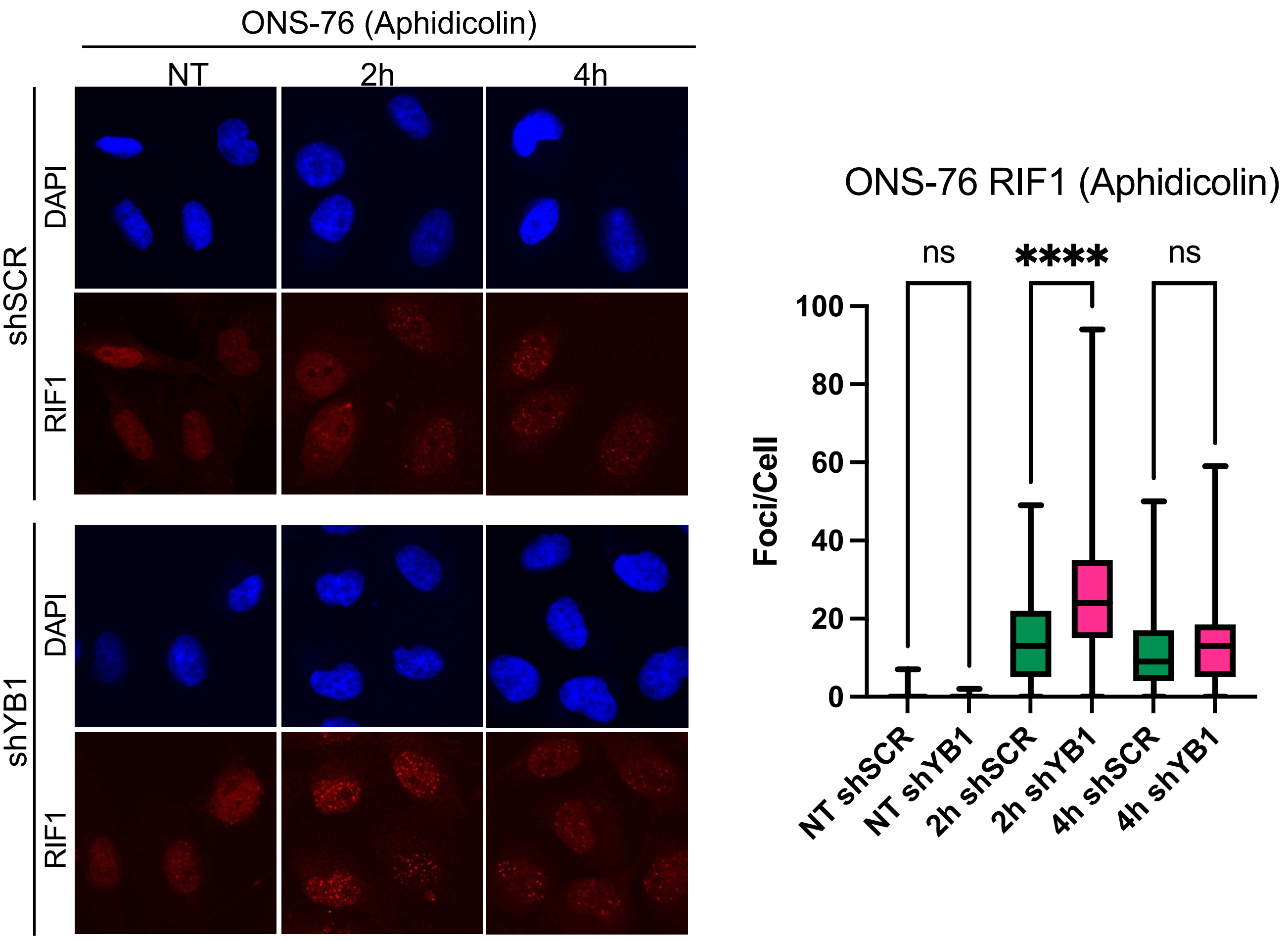

Supplementary Figure 9: 10Gy irradiation of S-phase synchronized ONS-76 shows greater RIF1 foci formation following YB1 depletion at 2 hours compared to control irradiated (2h mean rank diff. = -140.6 $p<0.0001$, 4h mean rank diff. = -47.75 $p=0.2346$, Kruskal-Wallis test n=3).

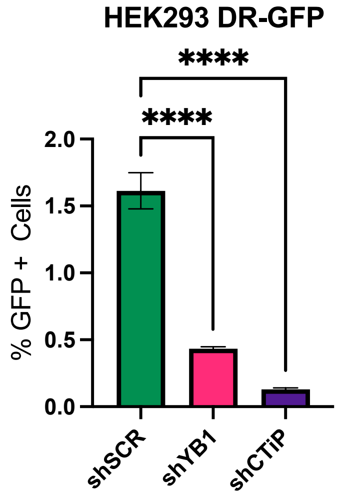

Supplementary Figure 10: (B) Three technical replicates of DR-GFP assay in 293T cells showing reduced induction of GFP in YB1 depleted cells following SCEI mediated cleavage compared to control with shCtIP as a positive control (shSCR vs shYB1 95% CI = 1.02-1.34 $p<0.0001$, shSCR vs shCTiP 95% CI = 1.32-1.65 $p<0.0001$, one-way ANOVA n=1).

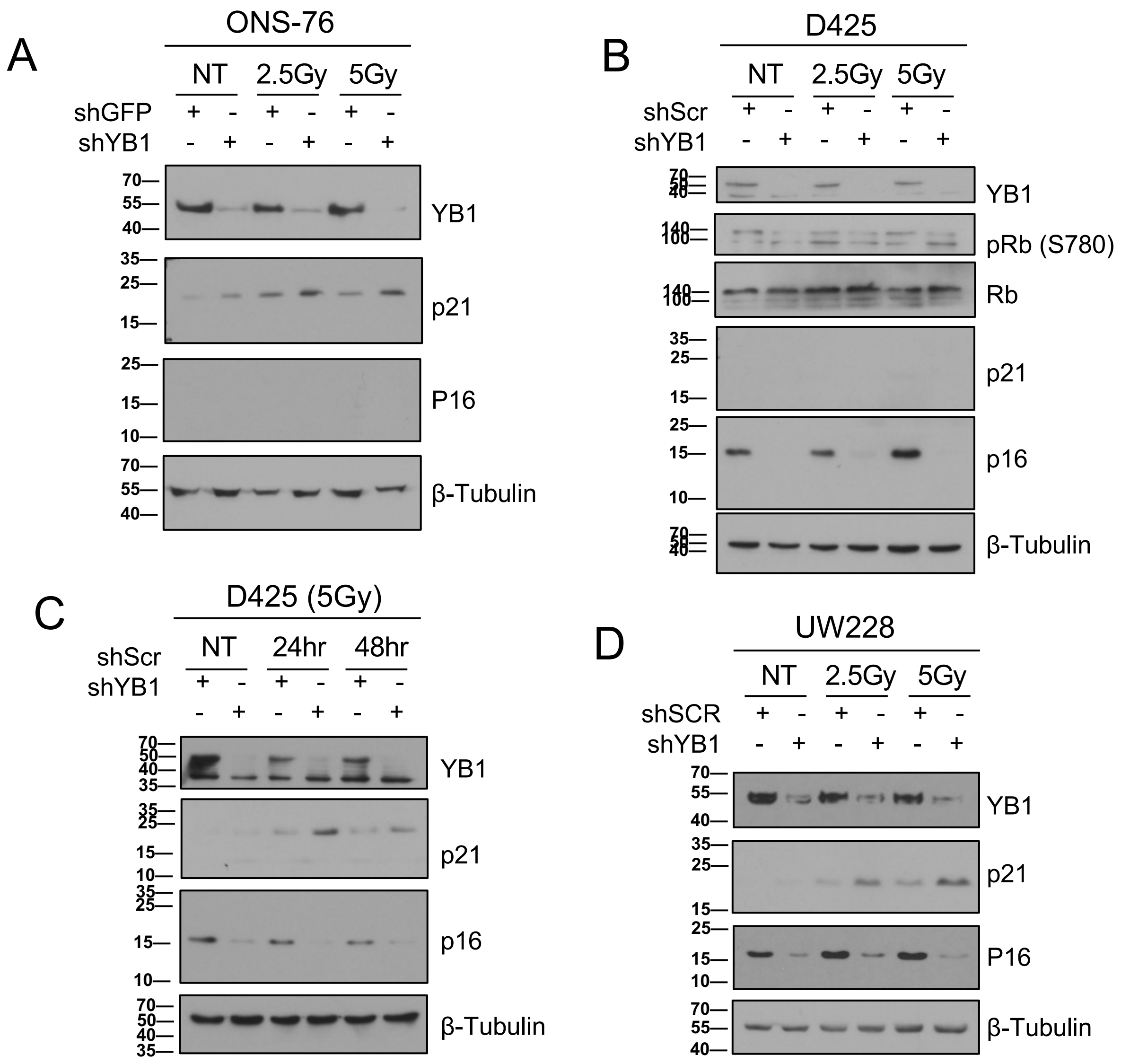

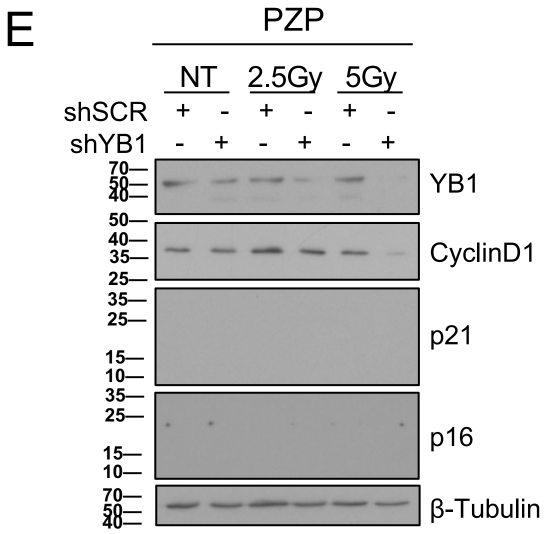

Supplementary Figure 11: (A) Blot of ONS-76 time-course (Figure 7) showing elevated p21 in irradiated YB1 depleted cells compared to control irradiated. **(B)** Blot of D425 time course (Figure 7) showing reduced proliferation (reduction of pRb) in irradiated YB1 depleted cells compared to control irradiated 5 days post radiation. **(C)** 5Gy irradiated D425 collected at 24 and 48 hours showing increased p21 in YB1 depleted cells. **(D)** Blot of UW228 (SSH MB p53mut) showing increased p21 in irradiated YB1 depleted irradiated cells compared to control irradiated 4 days post radiation. **(E)** Blot of PZP time course showing reduced proliferation (reduction of CyclinD1) in irradiated YB1 depleted irradiated cells compared to control irradiated 3 days following radiation.

ONS-76 Growth

Tukey's multiple comparisons test	Mean Diff.	95.00% CI of diff.	Summary	Adjusted P Value
shScr NT vs. shYB1 NT	439583	-43173 to 922340	ns	0.0891
shScr 2.5Gy vs. shYB1 2.5Gy	679167	196410 to 1161923	**	0.0026
shScr 5Gy vs. shYB1 5Gy	531250	48494 to 1014006	*	0.0251

ONS-76 Doubling Time

Tukey's multiple comparisons test	Mean Diff.	95.00% CI of diff.	Summary	Adjusted P Value
NT	-0.6633	-8.577 to 7.250	ns	0.9942
2.5Gy	-2.393	-10.31 to 5.520	ns	0.8034
5Gy	-13.45	-21.36 to -5.533	**	0.0015

PZP Growth

Tukey's multiple comparisons test	Mean Diff.	95.00% CI of diff.	Summary	Adjusted P Value
shScr NT vs. shYB1 NT	1333333	888467 to 1778200	****	<0.0001
shScr 2.5Gy vs. shYB1 2.5Gy	1087917	643050 to 1532783	****	<0.0001
shScr 5Gy vs. shYB1 5Gy	603333	158467 to 1048200	**	0.0039

PZP Doubling Time

Tukey's multiple comparisons test	Mean Diff.	95.00% CI of diff.	Summary	Adjusted P Value
NT	-0.6933	-1.688 to 0.3014	ns	0.2149
2.5Gy	-1.513	-2.508 to -0.5186	**	0.0036
5Gy	-4.11	-5.105 to -3.115	****	<0.0001

D341 Growth

Tukey's multiple comparisons test	Mean Diff.	95.00% CI of diff.	Summary	Adjusted P Value
shScr NT vs. shYB1 NT		0.3291 to 0.9692	*	0.045
shScr 2.5Gy vs. shYB1 2.5Gy		0.6798 to 0.8681	*	0.0114
shScr 5Gy vs. shYB1 5Gy		0.4117 to 0.9521	*	0.0407

D341 Doubling Time

Tukey's multiple comparisons test	Mean Diff.	95.00% CI of diff.	Summary	Adjusted P Value
NT	-11.28	-18.94 to -3.630	**	0.0074
2.5Gy	-20.94	-28.59 to -13.28	***	0.0001

D425 Growth

Tukey's multiple comparisons test	Mean Diff.	95.00% CI of diff.	Summary	Adjusted P Value
shScr NT vs. shYB1 NT	959167	744528 to 1173805	****	<0.0001
shScr 2.5Gy vs. shYB1 2.5Gy	594167	379528 to 808805	****	<0.0001
shScr 5Gy vs. shYB1 5Gy	257500	42862 to 472138	*	0.0124

D425 Doubling Time

Tukey's multiple comparisons test	Mean Diff.	95.00% CI of diff.	Summary	Adjusted P Value
NT	-48.52	-478.8 to 381.8	ns	0.978
2.5Gy	-507.3	-937.6 to -77.03	*	0.025

Supplementary Figure 12: List of all comparisons for Figure 7 showing Mean Difference, 95% confidence interval, and adjusted p value.

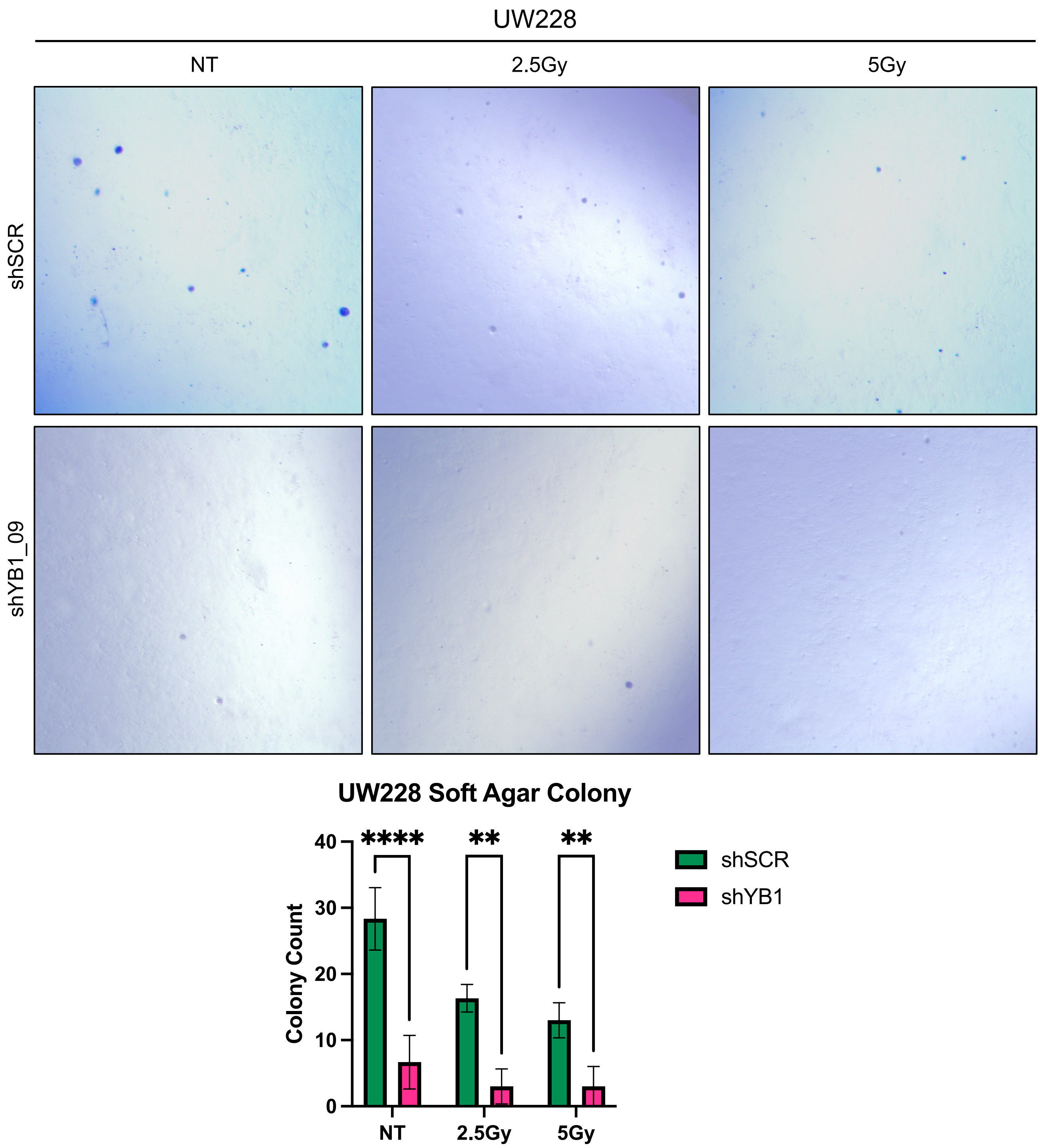

Supplementary Figure 13: Soft Agar Colony Formation assay of UW228 showing differences in colony formation that decreases proportional to the radiation dose (shSCR NT vs shYB1 NT 95% CI = 14.16-29.17 p<0.0001, shSCR vs shYB1 2.5Gy 95% CI = 5.831-20.84 p=0.0011, shSCR vs shYB1 95% CI = 2.498-17.50 p=0.0092 n=3 2-way ANOVA).

A

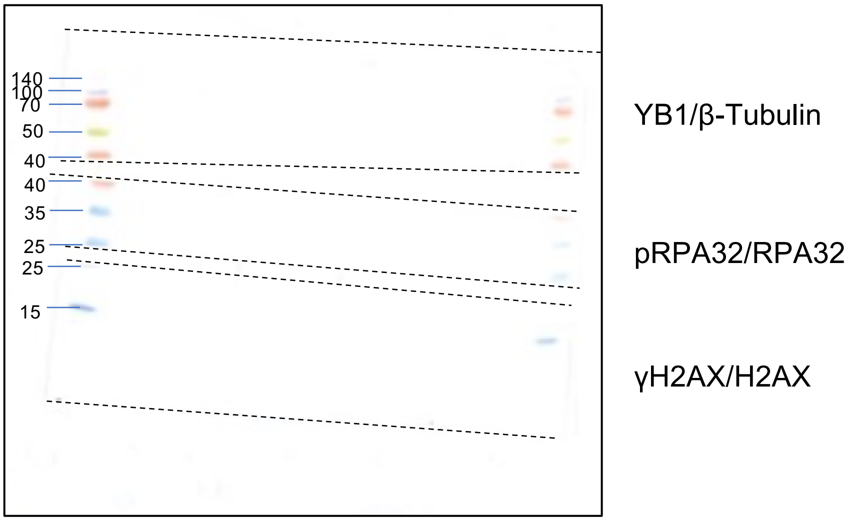

B

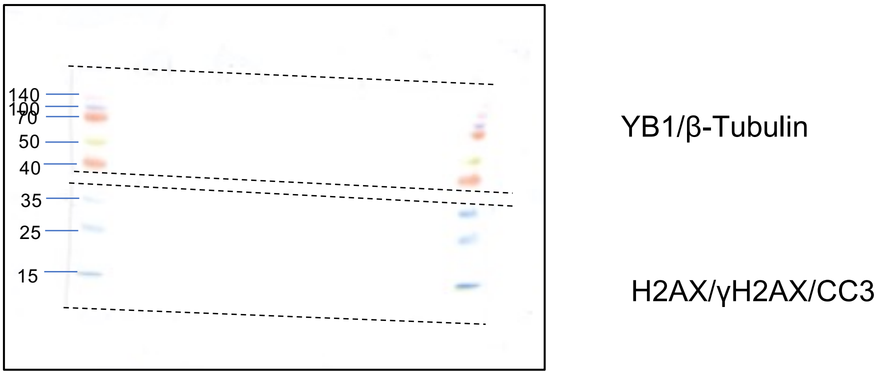

Immuno-blotting Supplementary Figure 1: (A) Blot with cutting pattern corresponding to Figure 4c, Supp Fig 6a-b and Supp Fig7. **(B)** Blot with cutting pattern corresponding to Figure 3D, Fig3A, Fig3D, Supp Fig 4A/B/D, and Supp Fig 7. For Supp Fig4a a separate blot was run for LaminB1 (See Immuno-blotting Supp Fig 2). Where multiple proteins are listed multiple blots were run. Blots are not stripped and re-probed.

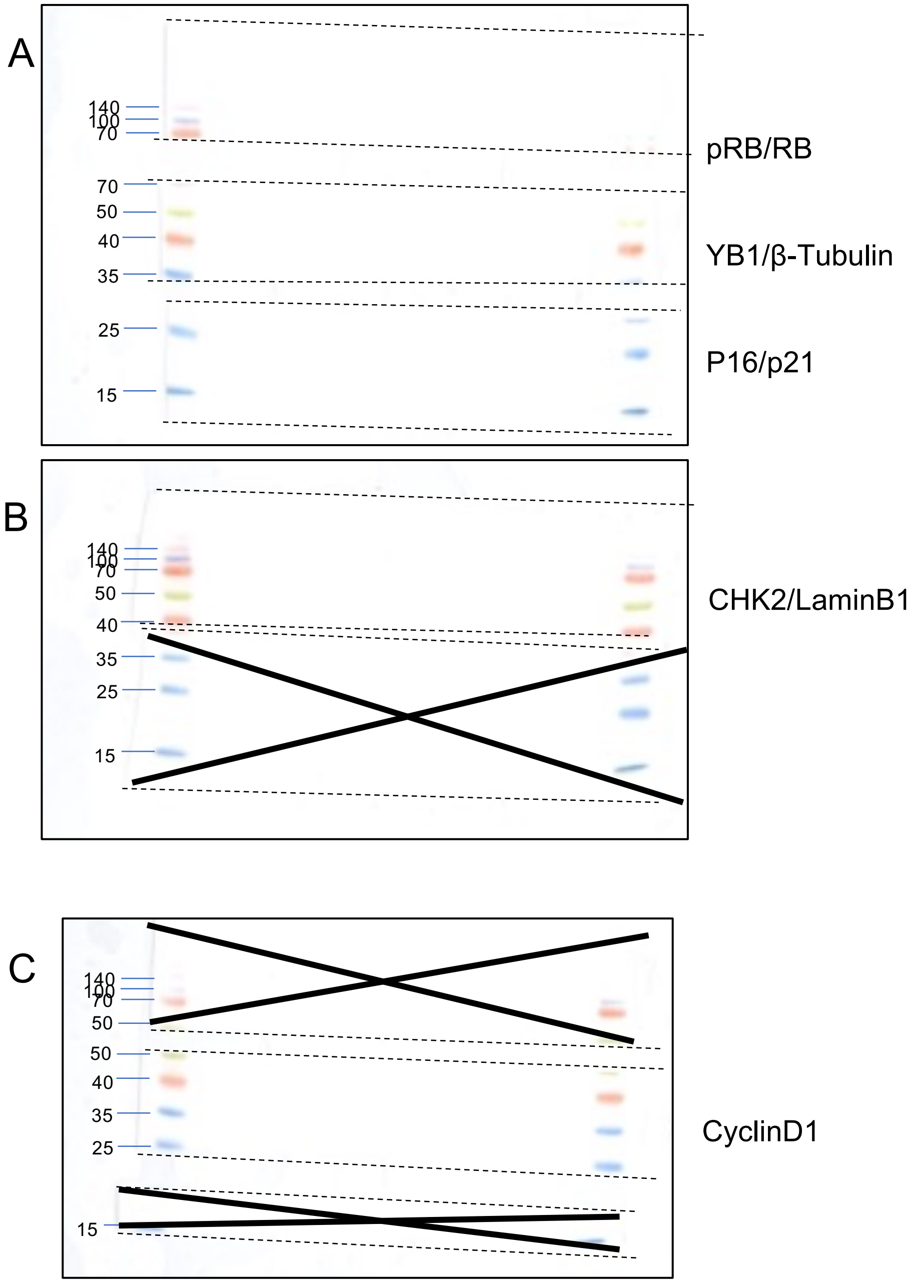

Immuno-blotting Supplementary Figure 2: (A) Blot with cutting pattern corresponding to Supp Fig 11a-e. (B) Blot with cutting pattern corresponding to Fig 3c (Chk2) and Supp Fig4a (laminB1). (C) Blot with cutting pattern corresponding to Supp Fig 11a. Blots segments with an X were disposed and not probed with antibody. Where multiple proteins are listed multiple blots were run. Blots are not stripped and re-probed.

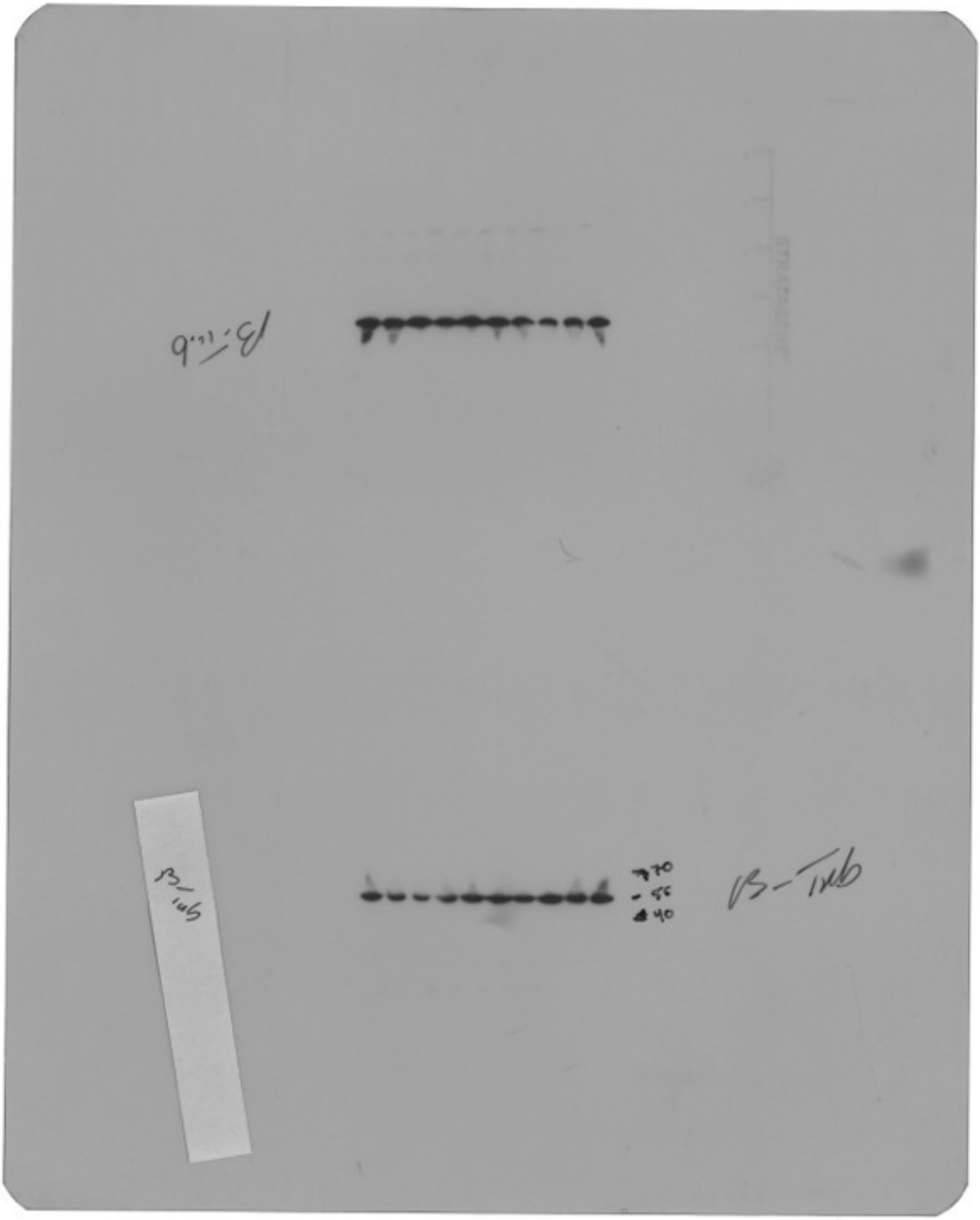

Immuno-blotting Supplementary Figure 3: representative auto-rad film **(**B-Tubulin) corresponding to Fig4c

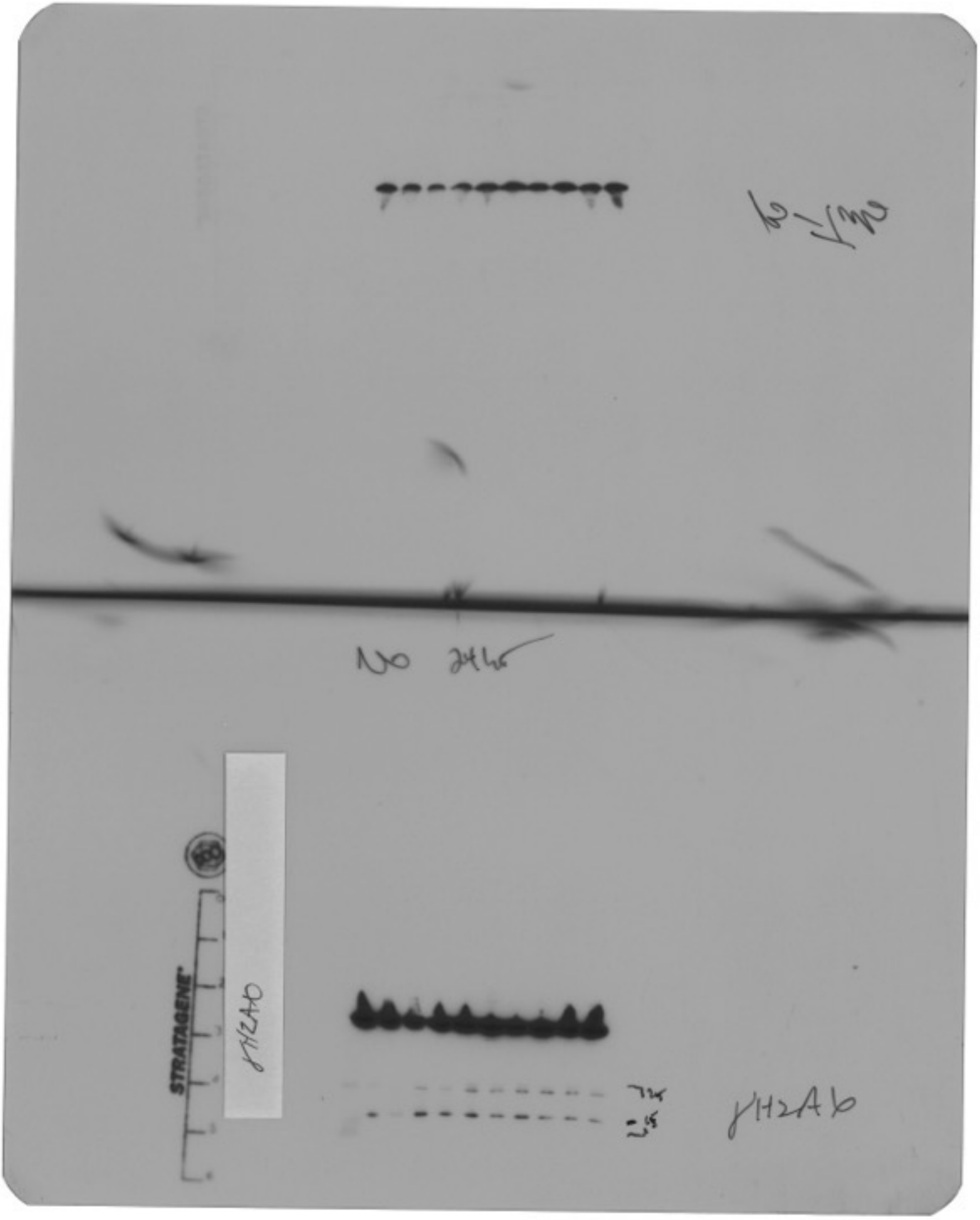

Immuno-blotting Supplementary Figure 4: representative auto-rad film (γH2AX) corresponding to figure 4c

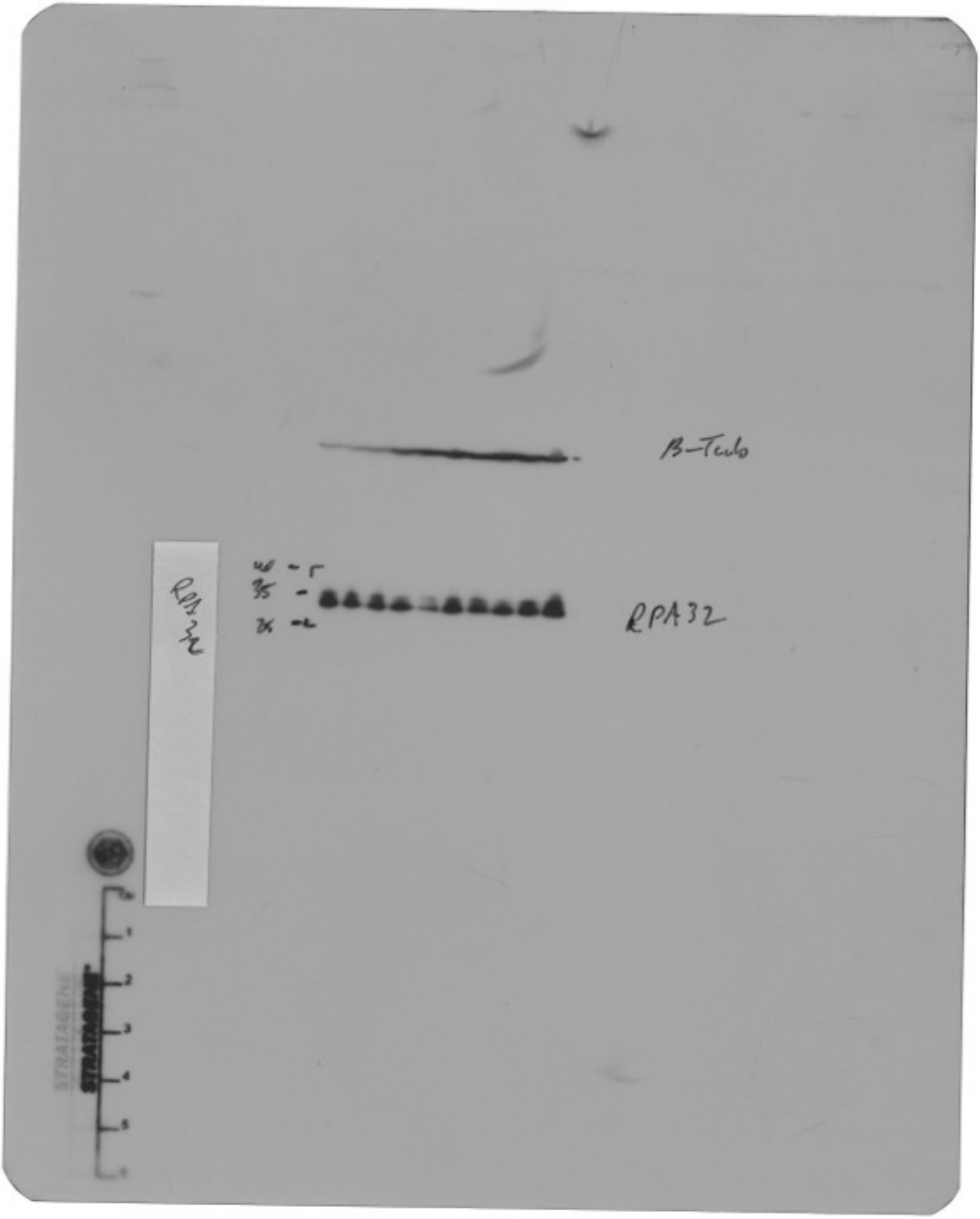

Immuno-blotting Supplementary Figure 5: representative auto-rad film (RPA32) corresponding to figure 4c

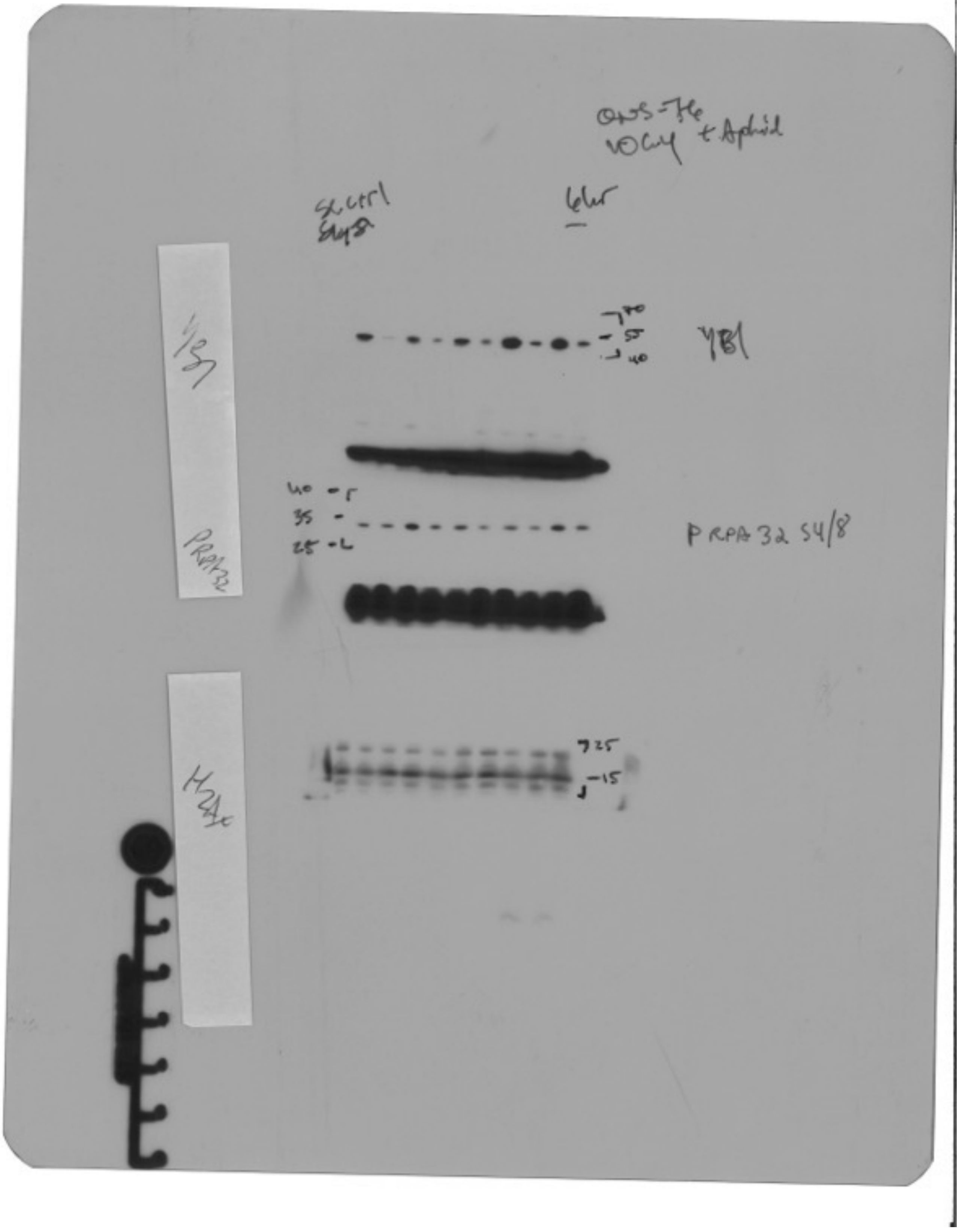

Immuno-blotting Supplementary Figure 6: representative auto-rad film (YB1 and pRPA32) corresponding to figure 4c
Note: H2AX was not utilized for this figure.

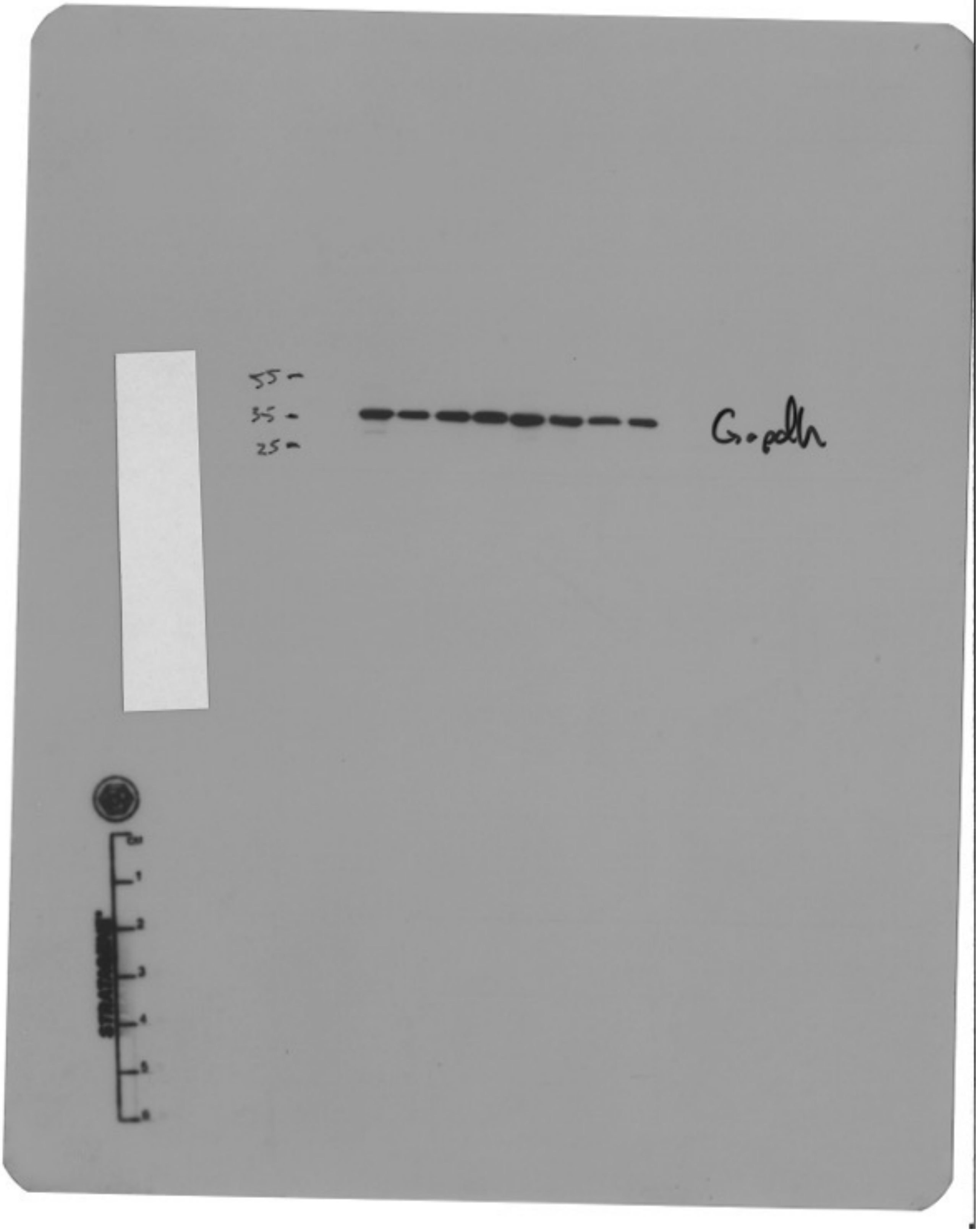

Immuno-blotting Supplementary Figure 7: representative auto-rad film (GAPDH) blot corresponding to Figure 1a bottom right
Note: only last 5 lanes used.

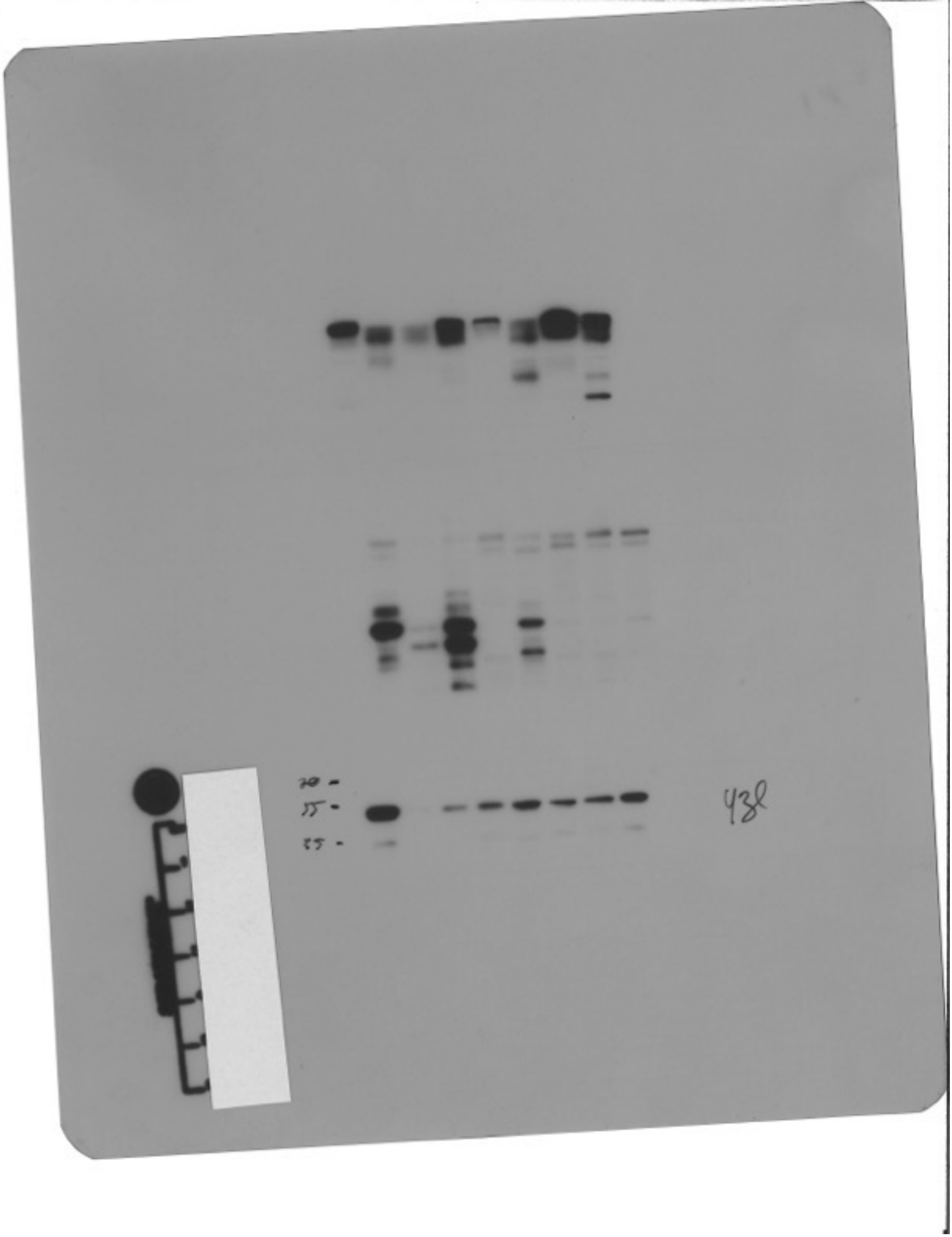

Immuno-blotting Supplementary Figure 8: representative auto-rad film (YB1) blot corresponding to figure 1a upper right
Note: only last 5 lanes included

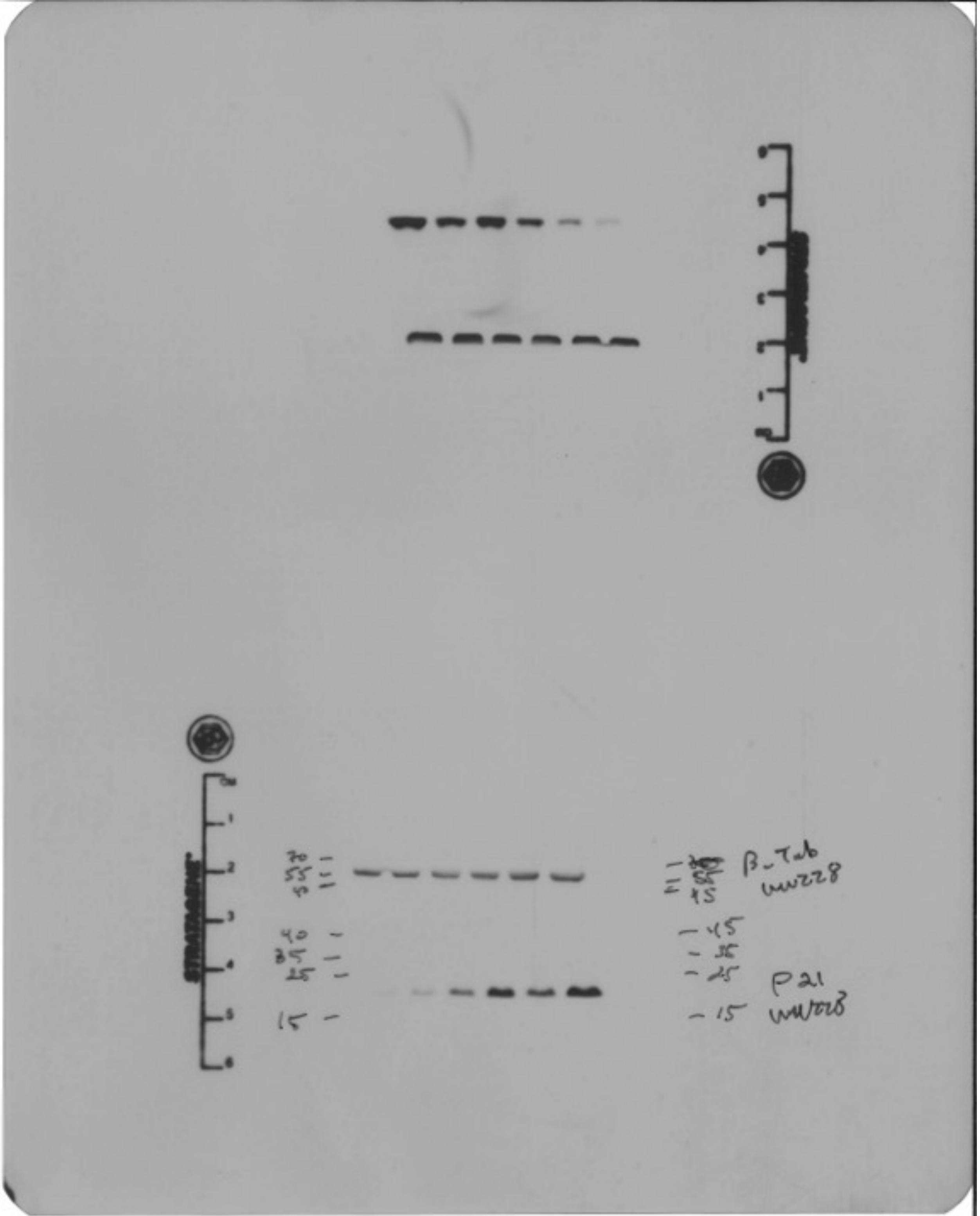

Immuno-blotting Supplementary Figure 9: representative auto-rad film (B-Tubulin) corresponding to supp figure 11d
Note: lighter exposure of p21 chosen for blot (See immuno-blotting supp fig 11)

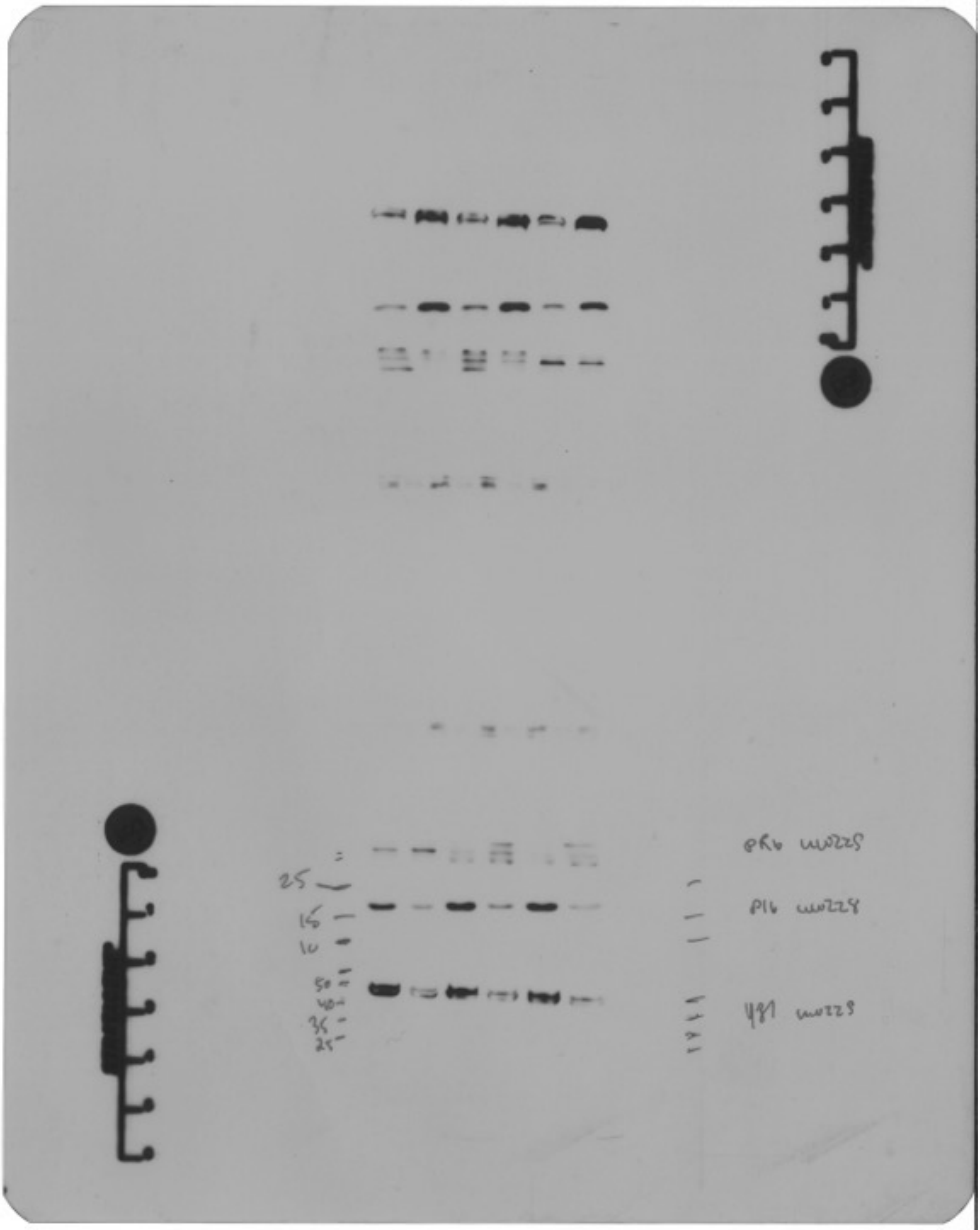

Immuno-blotting Supplementary Figure 10: representative auto-rad film (P16 and YB1) corresponding to supp figure 11d

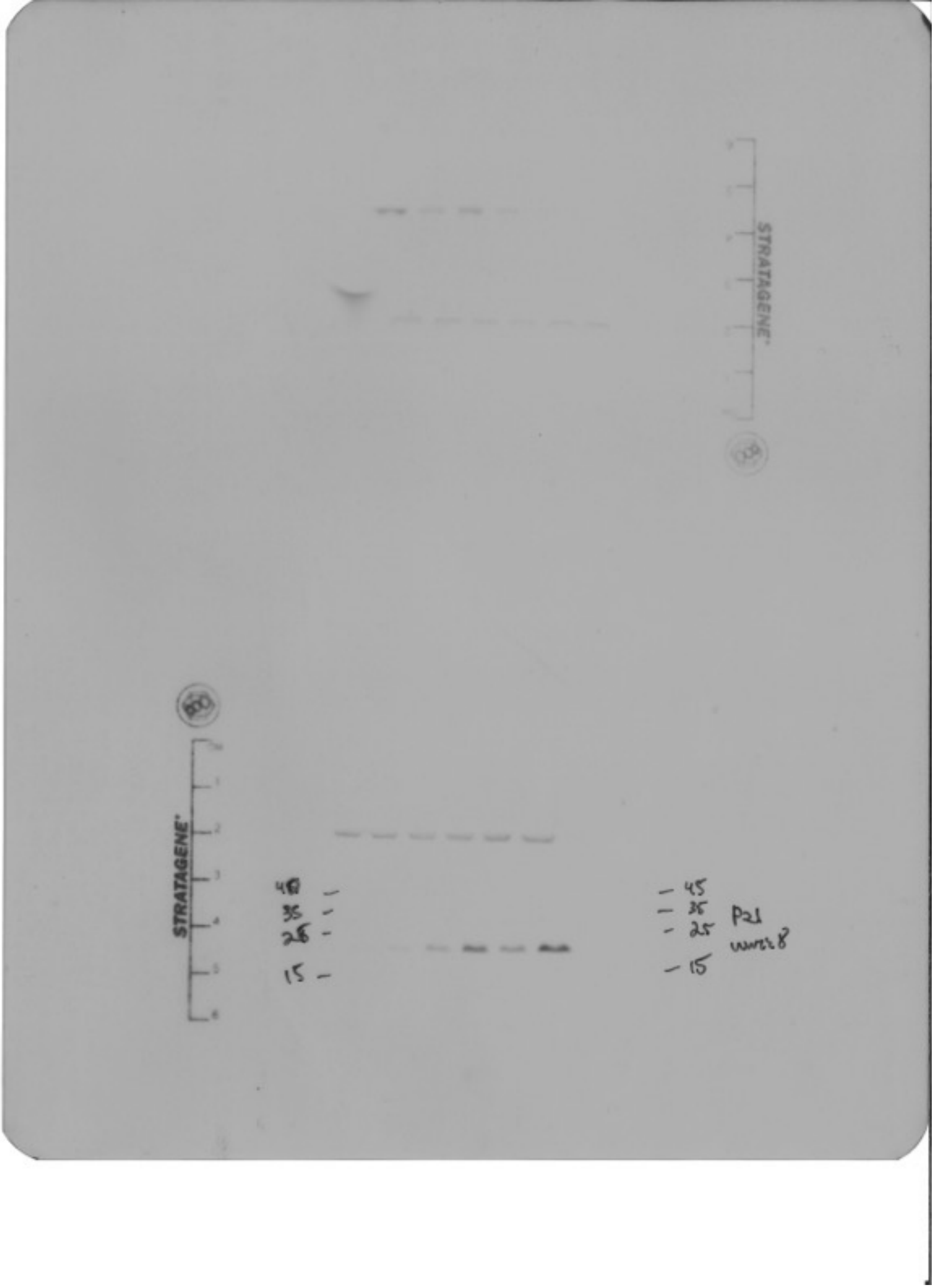

Immuno-blotting Supplementary Figure 11: representative auto-rad film (P21) corresponding to supp fig 11d

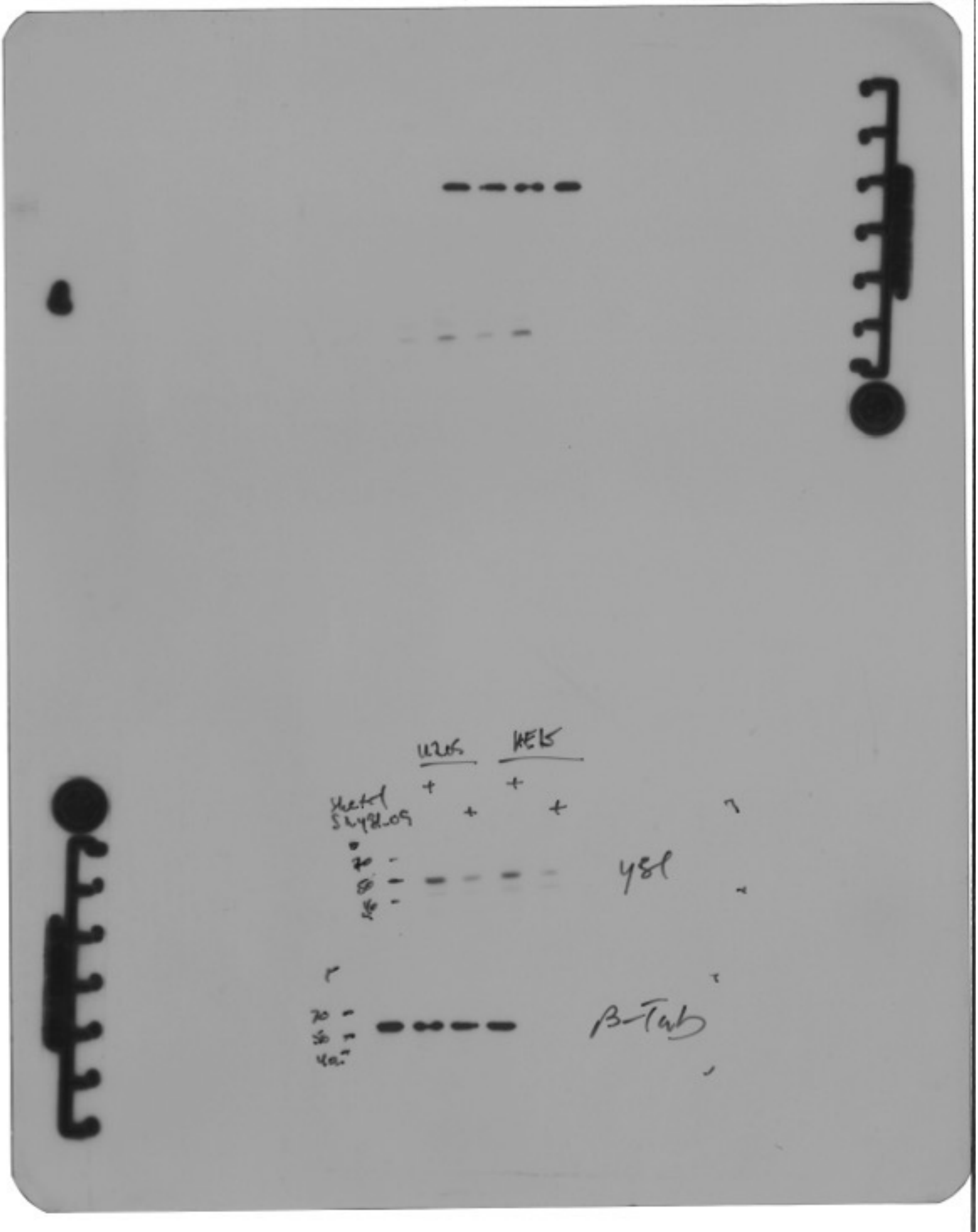

Immuno-blotting Supplementary Figure 12: representative auto-rad film (YB1 and β-Tubulin) corresponding to figure 6b

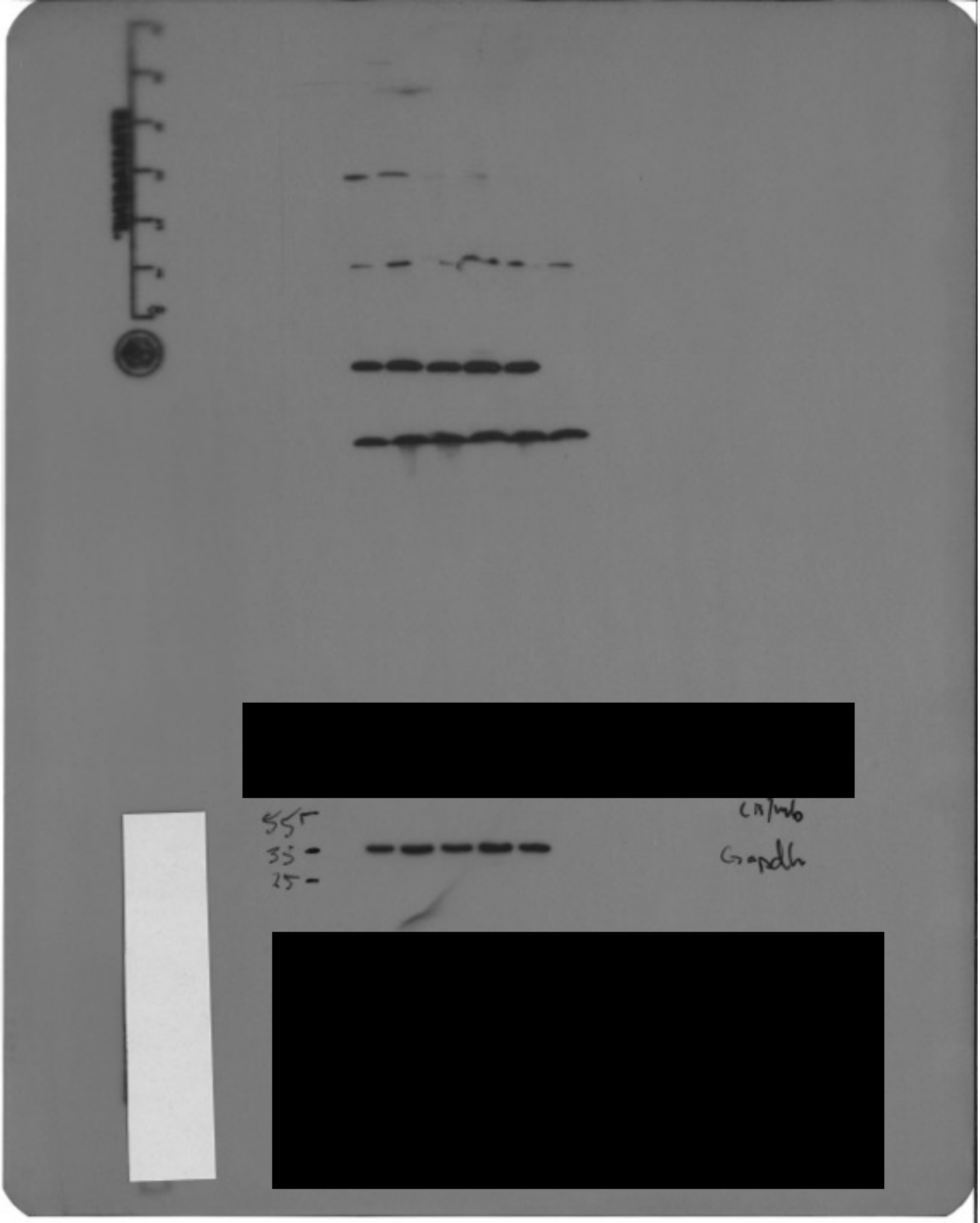

Immuno-blotting Supplementary Figure 13: representative auto-rad film (GAPDH) corresponding to figure 1a bottom left.
Black boxes excludes unpublished data unrelated to manuscript.

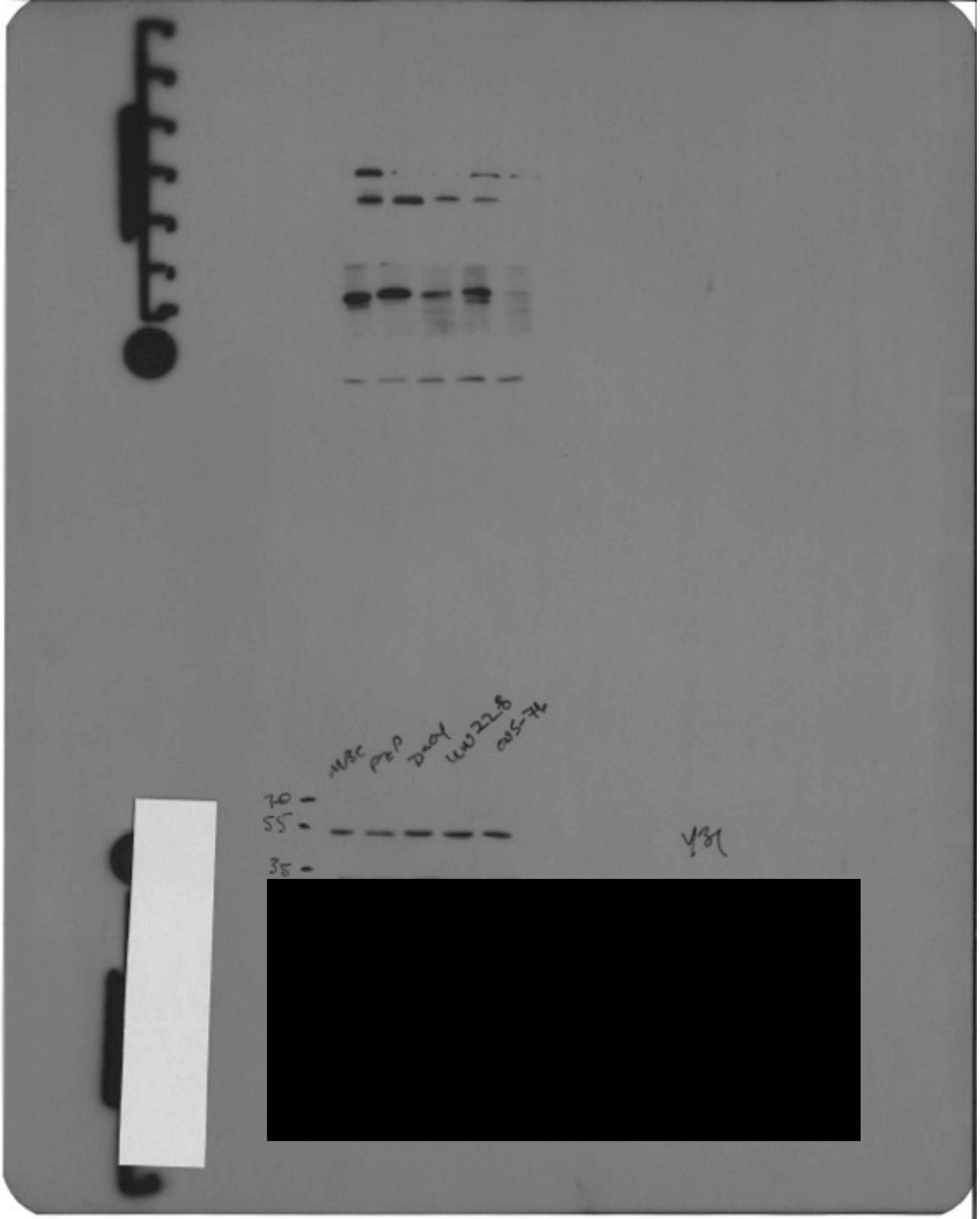

Immuno-blotting Supplementary Figure 14: representative auto-rad film (YB1) corresponding to figure 1a top right.
Black box excludes unpublished data unrelated to manuscript.

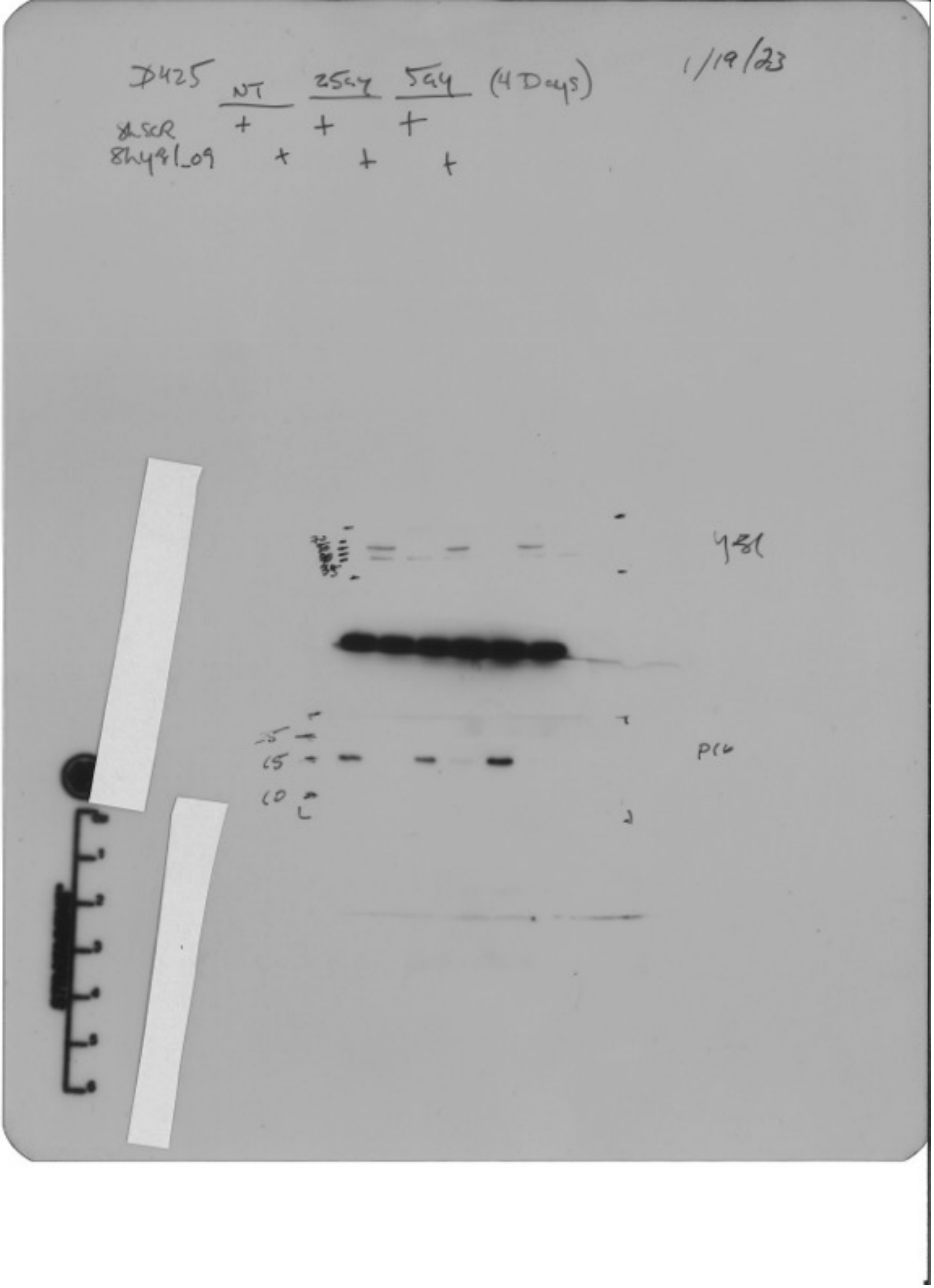

Immuno-blotting Supplementary Figure 15: representative auto-rad film (YB1 and p16) corresponding to supp figure 11b

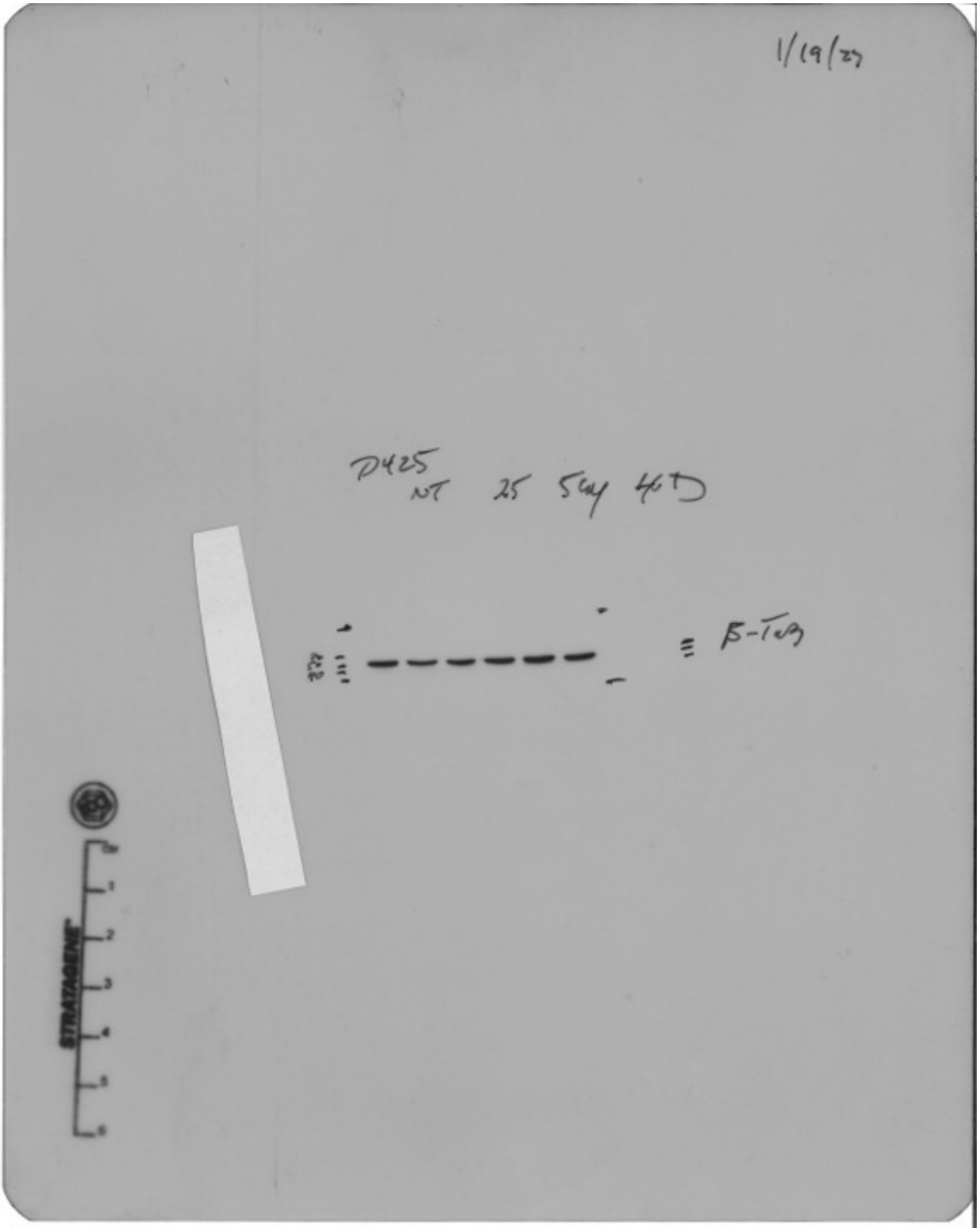

Immuno-blotting Supplementary Figure 16: representative auto-rad film (β-Tubulin) corresponding to supp figure 11b
